# The effects of electric power lines on the breeding ecology of greater sage-grouse

**DOI:** 10.1371/journal.pone.0209968

**Published:** 2019-01-30

**Authors:** Michel T. Kohl, Terry A. Messmer, Benjamin A. Crabb, Michael R. Guttery, David K. Dahlgren, Randy T. Larsen, Shandra N. Frey, Sherry Liguori, Rick J. Baxter

**Affiliations:** 1 Jack H. Berryman Institute, Department of Wildland Resources, Utah State University, Logan, Utah, United States of America; 2 Remote Sensing/GIS Laboratory, Quinney College of Natural Resources, Utah State University, Logan, Utah, United State of America; 3 Department of Plant and Wildlife Sciences, Brigham Young University, Provo, Utah, United States of America; 4 The Monte L. Bean Life Sciences Museum, Brigham Young University, Provo, Utah, United States of America; 5 Rocky Mountain Power/Pacific Power, Salt Lake City, Utah, United States of America; University of South Carolina, UNITED STATES

## Abstract

Anthropogenic infrastructure can negatively affect wildlife through direct mortality and/or displacement behaviors. Some tetranoids (grouse spp.) species are particularly vulnerable to tall anthropogenic structures because they evolved in ecosystems void of vertical structures. In western North America, electric power transmission and distribution lines (power lines) occur in sagebrush (*Artemisia* spp.) landscapes within the range of the greater sage-grouse (*Centrocercus urophasianus*; sage-grouse). The U.S. Fish and Wildlife Service recommended using buffer zones near leks to mitigate the potential impacts of power lines on sage-grouse. However, recommended buffer distances are inconsistent across state and federal agencies because data are lacking. To address this, we evaluated the effects of power lines on sage-grouse breeding ecology within Utah, portions of southeastern Idaho, and southwestern Wyoming from 1998–2013. Overall, power lines negatively affected lek trends up to a distance of 2.7 and 2.8 km, respectively. Power lines died not affect lek persistence. Female sage-grouse avoided transmission lines during the nesting and brooding seasons at distances up to 1.1 and 0.8 km, respectively. Nest and brood success were negatively affected by transmission lines up to distances of 2.6 and 1.1 km, respectively. Distribution lines did not appear to affect sage-grouse habitat selection or reproductive fitness. Our analyses demonstrated the value of sagebrush cover in mitigating potential power line impacts. Managers can minimize the effects of new transmission power lines by placing them in existing anthropogenic corridors and/or incorporating buffers at least 2.8 km from active leks. Given the uncertainty we observed in our analyses regarding sage-grouse response to distribution lines coupled with their role in providing electric power service directly to individual consumers, we recommend that buffers for these power lines be considered on a case-by-case basis. Micrositing to avoid important habitats and habitat reclamation may reduce the potential impacts of new power line construction.

## Introduction

Over the past century, human population growth has facilitated land use changes at a global scale [1]. The infrastructure (e.g., roads, electrical lines) required to support this human population growth can negatively impact wildlife [2]. Anthropogenic structures constructed in previously unfragmented landscapes [3], can have direct and indirect effects on local wildlife populations [4–6]. For example, tall anthropogenic structures such as power lines and wind turbines can cause direct mortality due to wildlife collisions [5, 7]. Anthropogenic development may also indirectly affect wildlife populations by causing changes in animal behavior (e.g., displacement [3]), increased predation risk [8], or by creating impediments to seasonal migration [9, 10]. The magnitude and consequences of these behavioral changes are often difficult to measure due to the absence of immediate population level effects [11, 12]. As such, identifying and quantifying both the direct and indirect effects of anthropogenic structures on wildlife as well as effective measures to minimize negative impacts are essential for conserving affected wildlife populations [3, 12].

The greater sage-grouse (*Centrocercus urophasianus*; sage-grouse) is a sagebrush (*Artemisia* spp.) obligate species that requires large, intact sagebrush systems [13]. Populations have declined nearly 50% in portions of their historic range [14]. Some of the observed declines have been attributed to the construction, operation, and maintenance of anthropogenic infrastructure such as tall structures and associated linear features (i.e., energy developments, roads and power lines) [15]. Some tetranoids, including sage-grouse, may be particularly vulnerable to tall anthropogenic structures because they evolved in landscapes void of such structures [3].

The potential incompatibility between tetranoids and anthropogenic structures led the U.S. Fish and Wildlife Service (USFWS) to identify the placement, operation, and maintenance of tall structures and associated linear features (i.e., roads and power lines) related to energy development and transmission in sagebrush habitats as a conservation threat for sage-grouse in their 2010 decision to designate the species as a candidate for protection under the Endangered Species Act of 1973 [16]. They concluded the placement of electric power lines in seasonal sage-grouse breeding habitats could impact local populations through increased predation of adults, juveniles, and nests or abandonment of suitable habitats.

The USFWS conclusion was based on research suggesting that oil and gas development can negatively influence sage-grouse lek counts, activity, survival, and habitat selection [17–21]. However, despite these and other studies (see UWIN [22]), it has been difficult to separate the combined influence of anthropogenic structures (e.g., well sites, roads) and their associated human activities from the presence of tall linear structures such as power lines [11, 23]. Furthermore, using studies of various types of energy development (i.e., oil/gas, wind) may not be comparable to power lines because of differing project footprints (polygons vs. linear) and amounts of human activity [11, 23]. For example, workers may be routinely present throughout oil fields or wind developments, yet power lines may only be visited infrequently for inspections or repairs. Power line inspection cycles vary depending on the line voltage, agency requirements, and location, but may occur as often as twice/year or as infrequent as once over five to ten years. Because power lines often occur on landscapes with other human activity, it is difficult to distinguish between potential impacts of the tall structures versus impacts related to other human activity, such as disturbance or habitat loss. In addition, isolating the effects of tall structures from other development activities on sage-grouse populations is particularly problematic if an a-priori status assessment was not completed before the structures were placed [22]. However, in the absence of experimental data, retrospective analysis of the potential effects of anthropogenic activities and their related structures on sage-grouse breeding distributions may provide new insights to prevent, minimize, or mitigate conservation threats [11].

Given that, our current knowledge regarding the influence of power lines on sage-grouse is limited and equivocal. Historically, researchers relied on untested causal mechanisms to infer the negative effects of power lines on sage-grouse [23]. For example, Knick et al. [24] used reported foraging distances of golden eagles (*Aquila chrysaetos*) [25, 26] and common ravens (*Corvus corax*) [8, 27] to estimate that electric power transmission lines had a negative impact on 50% of all sagebrush within the range of sage-grouse [11]. More recently, intense field studies have documented a reduction in sage-grouse habitat use [28–30] and vital rates [30, 31] in areas adjacent to power lines. Together, this has led to suggestions that power lines have been a potential factor in the extirpation of sage-grouse from historical ranges [32]. It is still unclear, however, under what conditions power lines negatively affect sage-grouse. For example, sage-grouse may not avoid power lines in years of low raven abundance [30]. There may also be site-specific conditions that counteract the negative influence of power lines on sage-grouse. This was demonstrated by Westover et al. [33] who observed positive selection for transmission lines during the brooding period in Utah. These temporally dependent and site-specific conditions may explain why Johnson et al. [34] did not detect a consistent relationship between distance to power lines and leks trends across the specie’s range.

This conflicting information has created a dilemma for wildlife and land managers who are attempting to develop best management practices (BMP) for power lines in sagebrush habitats. The USFWS and Avian Power Line Interaction Committee (APLIC) have recommended seasonal and spatial buffers as BMPs for power lines placed near leks in sage-grouse breeding habitats to minimize the potential for negative impacts [35, 36]. However, UWIN [20], Messmer et al. [11], and Manier et al. [37] concluded the BMP buffer zones vary widely (e.g., 0.3 to 8.0 km) because of challenges associated with interpreting the area influenced by power lines. In addition, differences between transmission and distribution lines, which serve different purposes and therefore can occur in different landscapes, and are constructed at differing heights, have not been previously assessed. Together, this lack of information, combined with the potential site-specific variation in sage-grouse response detailed above demonstrates the need for a landscape-scale evaluation of power line effects on sage-grouse.

Given this knowledge gap, the overarching purpose for our study was to provide an empirical evaluation of the effects of transmission and distribution power lines on sage-grouse breeding ecology and to identify appropriate buffer distances. Specifically, our objectives were to identify the relative effects of both transmission and distribution power lines on sage-grouse leks, seasonal habitat-use, and reproductive success. To achieve this, we analyzed Utah sage-grouse lek persistence rates, male lek counts, and radio-marked female sage-grouse breeding site selection, nest and brood success recorded from 1998–2013 in Utah, portions of southeastern Idaho, and southwestern Wyoming. These study populations represented the majority of sage-grouse populations in the study area including representation from all 13 Sage-Grouse Management Areas in Utah (> 30,164 km^2^). To our knowledge, this is the first landscape-level comparison for an entire jurisdictional entity (i.e., a US sate) examining the relationship between power line classification and known sage-grouse nest and brood locations. For comparison, other large-scale field studies that have evaluated the influence of power line on sage-grouse were ~ 3,400 km^2^ [28, 31] and 7,000 km^2^ [30]. Within this context, we hypothesized that transmission lines would have a larger effect on sage-grouse breeding ecology than distribution lines due to their increased structure height, which may provide increased perching and nesting habitat for avian predators, and larger right-of-way (ROW) corridors [38]. Similarly, distribution lines may not affect sage-grouse to the same extent as transmission lines because they are more homogenously distributed across the landscape, thus mitigating avoidance behaviors, and because they often occur in previously disturbed areas. This information will assist state and federal conservation planners to refine conservation BMP buffer zone recommendations for new power line construction to avoid and minimize the potential effects on sage-grouse and their breeding habitats.

## Materials and methods

### Study area

Our study area encompassed known sage-grouse breeding habitats and distributions in Utah, portions of southeastern Idaho, and southwestern Wyoming [39]. These habitats exhibited more natural and anthropogenic fragmentation when compared to the Wyoming Basin [40, 41]. Sage-grouse populations in the northern part of our study area inhabit sagebrush-steppe, while populations in central and southern Utah primarily use sagebrush semi-desert [42]. Both are shrub-dominated sagebrush systems contrasted with an increased herbaceous component in higher latitude sagebrush-steppe systems compared to lower latitude sagebrush semi-desert. Big sagebrush (*A*. *tridentata*) varieties typically dominate most landscapes with Wyoming (*A*. *t*. *wyomingensis*), basin (*A*. *t*. *tridentata*), and mountain (*A*. *t*. *vaseyana*) big sagebrush at lower, mid, and high elevations, respectively. Shallow soils support low (*A*. *arbuscula*) and black (*A*. *nova*) sagebrush communities throughout the study area.

### Sage-grouse data

The Utah Division of Wildlife Resources (UDWR) provided the 1998–2013 sage-grouse lek location and count data we used to conduct our lek persistence and lek trend analysis for the portion of the study area located in Utah (UDWR, unpublished data). We accessed the sage-grouse nest and brood location data used in our study from a geo-referenced database maintained by Utah State University (USU, unpublished data). This database contained > 17,000 sage-grouse Universal Transverse Mercator (UTM) locations recorded between 1998 and 2013 during radio-telemetry studies completed by researchers at USU and Brigham Young University (BYU) to describe sage-grouse breeding ecology [39]. This included 15 unique study areas that varied in study length (range = 2–15 years) dependent on management and conservation needs. To complete these studies, sage-grouse were captured, radio-marked with very high frequency (VHF) radio-collars, released on-site, and routinely monitored to assess vital rates and habitat-use using standard protocols [39, 43] described below. The study protocols were approved by the USU or BYU Institutional Animal Use and Care Committee (USU [2322, 2411, 2419, 2560, 1451, 2189, 942, 942R, 1194, 1404, 1332]; BYU [100302, 110301, 050301, 080402]). The UDWR approved Certificates of Registration permitting sage-grouse captures, radio-marking, and monitoring.

### Sage-grouse monitoring

We monitored radio-marked hens every few days until they began to localize in an area. Upon localization, we would located the hens approximately daily to verify nest initiation, which was determined when a female was found in the same location on two consecutive visits during the breeding season. Nesting was verified by visually locating the nesting females by walking concentric circles. Actively nesting females were observed 3–5 times per week until the nest hatched or failed. A successful nest hatch was determined when egg halves were found intact in or near the nest bowl, and/or the inner membrane of the eggs was separated from the shell [44]. A failed nest was determined when no eggs or egg halves were found at the nest site, if egg halves were not intact, or if only egg fragments remained at or near the nest site [45]. We attempted to verify nest fate by locating the marked hen and recording brooding type behavior or visually identifying chicks as soon as the nest had been vacated. A global positioning system (GPS) location was recorded at each nest after fate was determined and the female was no longer present.

After a successful hatch, we attempted to locate broods two to three times per week until the brood reached 42 days of age or until the brood failed. We tracked brooding females using radio-telemetry, and walked concentric circles around the female location until brooding behavior or chicks were observed. Upon brood confirmation, we recorded a GPS location where the female was observed. A failed brood was determined if the female flushed with one or more adult females and no chicks were seen on two consecutive location attempts.

### Landscape variables

#### Distance to power lines

We acquired geo-referenced linear electric power distribution and transmission line (power line) locations directly from the electric utility companies PacifiCorp, Garkane Energy, Idaho Power, and Raft River Rural Electric Cooperative. These data were provided through confidentiality agreements specifically for this study. Each line in the power line database contained attributes indicating its status as a distribution line (<46 kV) or as a transmission line (> = 46 kV); in total the data represent 16,493 km of transmission lines and 20,061 km of distribution lines throughout Utah, southern Idaho, and southwestern Wyoming.

Transmission lines, which deliver electric power from the source of generation to substations, are supported by taller structures (height: 18–40 m). These lines included linear structures that were located at lower elevations and often bisected sage-grouse habitat. In contrast, distribution lines are supported by shorter structures (height: ~ 10 m) that deliver electric power to customers (e.g., homes, businesses). Distribution lines were interspersed throughout sage-grouse habitat [22]. All power lines evaluated were in service prior to 1998.

**Fig 1:**
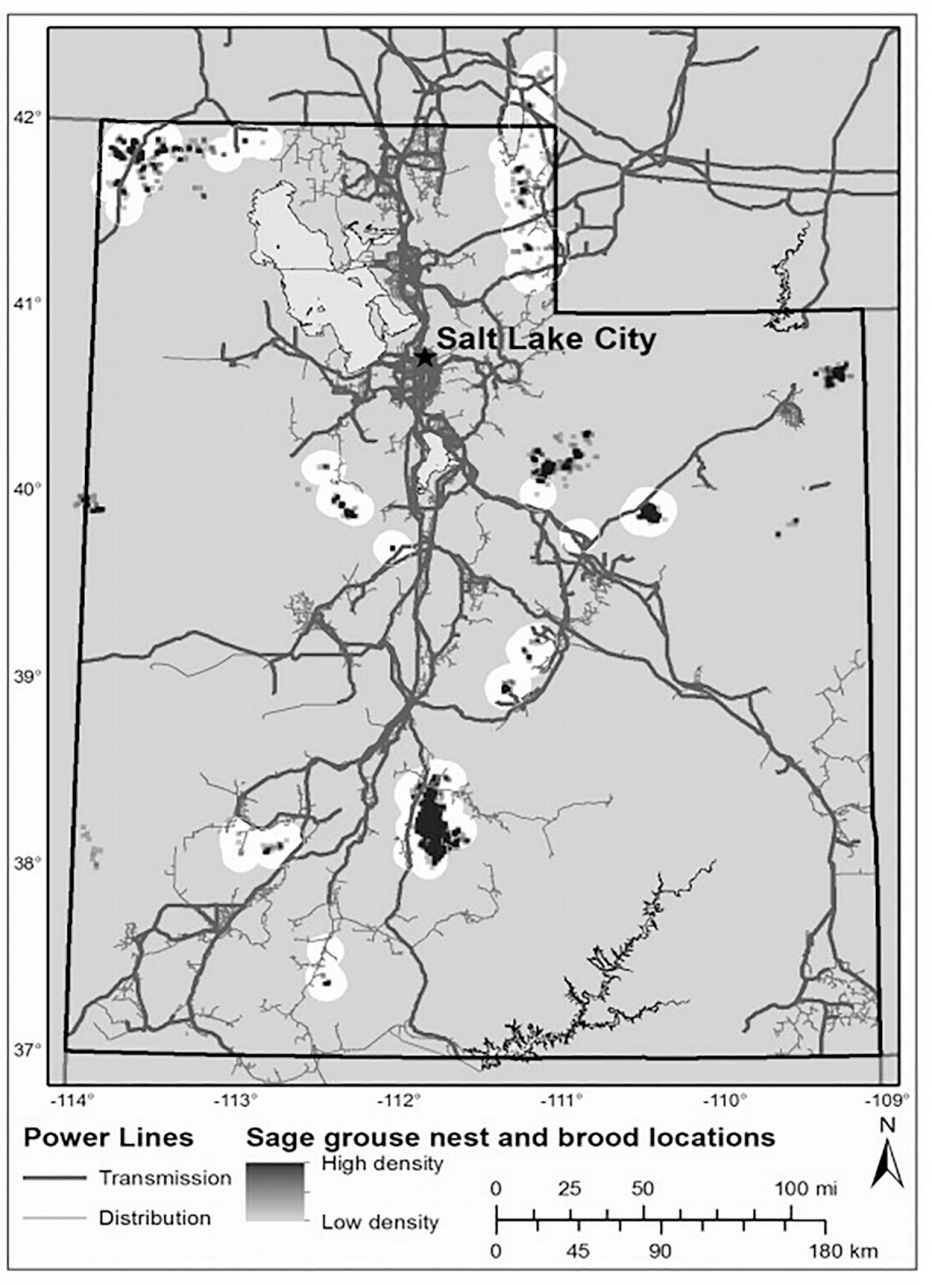
Electric transmission and distribution power lines relative to greater sage-grouse (*Centrocercus urophasianus*; sage-grouse) nest and brood locations in Utah, portions of southeastern Idaho, and southwestern Wyoming, USA, 1998–2013. Highlighted areas indicate sage-grouse locations within 10 km of the power lines studied.

We restricted our analysis to power lines located in occupied sage-grouse habitat [39] and excluded all lines that were not within 10 km of a sage-grouse lek, nest or brood location (Fig 1). We selected this buffer distance because it accommodated the average maximum movement distances documented for sage-grouse broods from nest sites in Utah (8.45 km) [39] and the upper recommended conservation buffer zones reported in the literature for tall structures and linear features (8.0 km) [37]. Moreover, assessing data, and as such, buffers beyond 10 km is unlikely to contribute to sage-grouse conservation because most sage-grouse habitat falls within 8 km of a lek [37]. Consequently, only 10% of transmission lines and 7% of distribution lines within the study area were within 10 km of these sage-grouse use habitats. This resulted in a reduced dataset of 1,698 km and 1,496 km of electric power transmission and distribution lines, respectively (Fig 1), and a sample of 425 nests and 2,514 unique brood locations obtained from 239 broods. We then constructed distance from transmission and distribution power lines using the spatstat library [46] in R (Version 3.3.3; [47]).

#### Roads

Because power lines can often parallel roads, we determined the percentage of the power lines adjacent to roads using road location data obtained from the U.S. Census Bureau [48]. Most of the electric power distribution (69% and 95%) and transmission (54% and 87%) lines in our study area were located within 100 m and 500 m of a road, respectively. When we considered distribution lines within 10km of a sage-grouse location, 69% were within 100 m of a road and 95% were within 0.5 km of a road. When we considered transmission lines within 10 km of a sage-grouse location, 54% were within 100 m of a road and 87% were within 0.5 km of a road. Because over 90% of the power lines analyzed were within 0.5 km of a road and roads frequently bisected power lines, we did not have a robust sample size to isolate differences between power lines with and without roads.

#### Percent sagebrush cover

To assess the percent of sagebrush cover, we quantified the proportion of 30-m resolution pixels classified as any type of sagebrush within 1 km of a given pixel using the moving window analysis in ArcGIS 10.3. The ‘Sagebrush’ category represents the combination of nine shrubland cover types identified by LANDFIRE 1.2.0 System Group physiognomies [40] and considered suitable sage-grouse habitat [40]. These communities included; 1) Colorado Plateau, 2) Mixed Low Sagebrush Shrubland, 3) Wyoming Basins Dwarf Sagebrush Shrubland and Steppe, 4) Great Basin Xeric Mixed Sagebrush Shrubland, 5) Inter-Mountain Basins Big Sagebrush Shrubland, 6) Columbia Plateau Low Sagebrush Steppe, 7) Inter-Mountain Basins Big Sagebrush Steppe, 8) Inter-Mountain Basins Montane Sagebrush Steppe, 9) Inter-Mountain Basins Semi-Desert Shrub-Steppe, and 10) *A*. *tridentata* ssp. *vaseyana* Shrubland Alliance.

#### Elevation

Because broods have been shown to move up in elevation as the season progresses ([39]), we included a measure of elevation in our analyses. This data (30 x 30 m) was obtained from the National Elevation Dataset administered by the U.S. Geological Survey (https://www.usgs.gov/core-science-systems/ngp/3dep).

### Data analysis

We tested for the influence of power lines on lek trends, lek persistence, nest and brood site selection, and nest and brood success. For each analysis, we developed a linear model that tested the relationship between distance to power lines and the response metric of interest (e.g., lek trend). We then compared this model to a null model to establish whether a power line effect existed. We next added measures of sagebrush cover, elevation, and distance to roads as covariates of interest to assess whether these covariates improved model fit. Lastly, we tested for an interactive effect between distance to power lines and our measure of sagebrush cover because previous work has identified this as a potential driver of sage-grouse habitat selection and vital rates [30]. Due to model complexity, we did not assess other interactions. In total this produced 13 model sets (Table 1).

**Table 1:** Model combinations used to evaluate the influence of power lines on sage-grouse (*Centrocercus urophasianus*) habitat selection and vital rates.

Model Sets		Lek Trend	Lek Persistence	Nest Selection	Brood Selection	Nest Success	Brood Success
Null Model							
	Null	✓	✓	✓	✓	✓	✓
Linear Models							
** **	DistTL	✓	✓	✓	✓	✓	✓
** **	DistTL + Sage			✓	✓	✓	✓
** **	DistTL + Elev			✓	✓	✓	✓
** **	DistTL + Rd			✓	✓	✓	✓
** **	DistTL + Sage + Elev			✓	✓	✓	✓
** **	DistTL + Sage + Rd			✓	✓	✓	✓
** **	DistTL + Elev + Rd			✓	✓	✓	✓
** **	DistTL + Sage + Elev + Rd			✓	✓	✓	✓
** **	DistTL * Sage			✓	✓	✓	✓
** **	DistTL * Sage + Elev			✓	✓	✓	✓
** **	DistTL * Sage + Rd			✓	✓	✓	✓
	DistTL * Sage + Elev + Rd			✓	✓	✓	✓
Nonlinear Models							
** **	DistT1 + DistT2	✓	✓	✓	✓	✓	✓
** **	DistT1 + DistT2 + Sage			✓	✓	✓	✓
** **	DistT1 + DistT2 + Elev			✓	✓	✓	✓
** **	DistT1 + DistT2 + Rd			✓	✓	✓	✓
** **	DistT1 + DistT2 + Sage + Elev			✓	✓	✓	✓
** **	DistT1 + DistT2 + Sage + Rd			✓	✓	✓	✓
** **	DistT1 + DistT2 + Elev + Rd			✓	✓	✓	✓
** **	DistT1 + DistT2 + Sage + Elev + Rd			✓	✓	✓	✓
** **	DistT1 * Sage + DistT2 * Sage			✓	✓	✓	✓
** **	DistT1 * Sage + DistT2 * Sage + Elev			✓	✓	✓	✓
** **	DistT1 * Sage + DistT2 * Sage + Rd			✓	✓	✓	✓
	DistT1 * Sage + DistT2 * Sage + Elev + Rd	** **	** **	✓	✓	✓	✓

* Sage represents our percent sagebrush cover covariate, Elev represents an elevation covariate, and Rd represents a distance to any road covariate.

Check marks identify whether a model set was evaluated for a given response metric.

Next we tested for a threshold effect of distance to power lines on the response metric of interests (e.g., lek trend). Within this context, we hypothesized that if distance from a power line affected sage-grouse, the effects would be magnified for sage-grouse that were located closer to power lines [11, 24, 37]. Thus, to explicitly evaluate the distance at which sage-grouse response to power lines changed, we tested for a response threshold by comparing the aforementioned model combinations (Table 1) to models with a threshold effect specified by two piecewise linear splines. Piecewise splines consist of a continuous covariate (e.g., distance to power line) defined over specified segments (e.g., > and < 2.0 km) and a response variable (e.g., lek trend) that is a continuous function of the covariate over all segments but with different slopes in each of the segments. To determine the presence and placement of thresholds, we performed a grid search of candidate models that included the sequential placement of a threshold every 0.1 km from 0.1–9.9 km. To minimize outlier effects that may bias threshold locations, we limited the threshold location such that it fell within the 5% and 95% quantiles of data for each analysis. This resulted in between 85 and 92 grid search candidate models depending on the analysis.

We tested for the additive (percent sagebrush cover, elevation, and distance to roads) and interactive effects (power lines x percent sagebrush cover) on each response metric of interest as described above. This produced 12 additional model sets which we evaluated (Table 1). Due to convergence issues, we were not able to assess the importance of additional covariates (i.e., percent sagebrush cover, distance to roads, elevation) for lek persistence and lek trends. Across all model sets (n = 24; Table 1), we selected the model with the lowest Akaike Information Criterion corrected for small sample sizes (AICc; [49]) score as our best fitting model. However, if a threshold model demonstrated equal support (ΔAICc ≤ 2.0) as being the best-fit model to either a null or linear model, we also highlighted this information in our results because this directly addressed our primary interest in identifying any response thresholds relative to power lines.

If a threshold model was identified as the best-fitting model, we then identified uncertainty (i.e., 95% confidence intervals) around the placement of each threshold. For general linear models (GLM), we used the ‘confint.segemented’ command within the segmented library in R. This approach was used for the lek trend and lek persistence analyses. Because the segmented library did not accommodate the use of generalized linear mixed models (GLMM), we identified uncertainty around a threshold GLMM as any threshold model that fell within ΔAICc of 2.0 of the best-fit threshold model [50]. This uncertainty approach was applied to the nest selection, brood selection, nest success, brood success analyses.

Although piecewise linear splines are useful for identifying thresholds, the segmenting of data leads to reduced sample sizes for a given spline. This contributes to a lack of statistical significance due to the reliance of p-values on sample size [51]. As such, we ignored arbitrary significance thresholds (e.g., p-value ≤ 0.05) in our interpretation of power line effects [52, 53], and rather, focused our interpretation on the marginal response of sage-grouse to power lines (i.e., slope of the relationship). As such, we do not provide measures of significance (i.e., p-values) in our model outputs, but rather present only information on confidence intervals for each model coefficient.

#### Preponderance of evidence approach

Many of our analyses consisted of small sample sizes, which when combined with likely population differences in habitat selection and vital rates across study sites, likely contributed to increased model uncertainty regarding the influence of power lines on our response variables of interest. Thus, we evaluated each response metric individually within the results section, and then used a proponderance of evidence approach [54] to assess whether our landscape scale analyses was suggestive of a power line effect. More specifically, we evaluated the relationship between distance to power lines and our response metric for each analyses using the best-fit model. Then, in our weight of evidence approach, we examined additional models sets that had equal support as the best-fit model (≤ ΔAICc of 2.0) to assess whether a threshold effect may exist. For example, if a linear model was the best-fit model (ΔAICc of 0.0) but a piecewise spline model was within ΔAICc of 2.0, we interpreted the result as having potential support for a threshold effect, and then examined whether or not that potential support existed across analyses. This interpretation was based on the marginal response coefficients for the spline prior and after the threshold for the best-fitting nonlinear model. If a model coefficient was positive prior to the threshold, we interpreted that as support for a negative power line effect at distances close to the power line.

### Lek trends

We employed a 2-stage analytic approach to assess the impact of distance to power lines on sage-grouse lek trend. In the first stage we estimated the overall count trend for each lek. In the second stage we treated the estimated trends as our response variable and modeled them as a function of distance to the nearest power line using both a continuous linear effect as well as piece-wise linear trends to determine if the magnitude or direction of the effect changed at some distance.

Statewide, 379 leks were monitored in ≥ 1 year between 1998 and 2013. However, to obtain reliable lek trend estimates we only analyzed data from leks that were monitored in ≥ 12 years, thereby reducing the total number of leks retained for analysis to 172 leks. The temporal trend for each lek was estimated using a Negative Binomial generalized additive model (GAM) to model the annual maximum number of males counted for lek *i* in year *t* as a linear function of year while accounting for population cycles [50] by fitting a smoothed effect of the total number of males counted statewide in each year. To be comparable through time, the annual total number of males was calculated using only those leks that were monitored in all 16 years of the study period. A negative binomial distribution was used because many leks had an excess number of zero males counted, thereby violating the mean-variance relationship of the Poisson distribution. Despite having reduced the dataset to only include those leks that were monitored in ≥ 12 of the 16 years, the trend analysis for some leks produced warning messages suggesting that the models did not converge and parameter estimates were not reliable. Concomitantly, we further reduced the resulting vector of estimated lek trend estimates by retaining only those estimates that were within 1 standard deviation of the average trend estimate. This resulted in a sample size of 125 lek trend estimates being carried forward to the second stage of our analysis.

To examine the role of power lines on lek trends, we constructed a General Linear Model with a Gaussian distribution as implemented in the base R (Version 3.3.3; [47]). To evaluate the potential for a response threshold, we used the aforementioned grid search approach to compare the linear and nonlinear model fits. The small number of leks for which we had trend estimates and were within 10 km of either transmission or distribution lines (n = 70) required that we analyze lek trends as function of distance to combined power line types. Distance to power lines (km) was included as the sole explanatory variable because the small number of leks precluded the evaluation of other habitat variables (e.g., percent sagebrush cover, roads) due to convergence issues.

### Lek persistence

We defined persistence as leks where at least 2 strutting males were observed in 2 years of each of the 5-year periods 1996–2000 and 2009–2013 [55, 56]. To examine the role of power lines on lek persistence, we constructed a GLM with a binomial distribution (persist vs. not persist) and logit-link function as implemented in the base R package. We employed the grid search of potential response thresholds similar to the lek trend analysis to compare the linear and nonlinear model fits. Due to the small number of leks within 10 km of either transmission or distribution lines (n = 56), we analyzed lek persistence as the function of distance to combined power line types. Distance to power lines (km) was included as the sole explanatory variable because the small number of leks precluded the evaluation of other habitat variables (e.g., percent sagebrush cover, roads) due to convergence issues.

### Nest and brood site selection

We used a resource selection function (RSF) approach within a use-available study design [57] evaluated at the population level [58] to determine the relative influence of distance to power lines and sagebrush cover on sage-grouse nest or brood-site selection. We included sagebrush cover because it has previously been reported as a factor that influences sage-grouse distributions [24, 59].

We used our database of known sage-grouse nest and brood locations and randomized potential locations with our study area to conduct the RSF. For the nest RSF, we generated 15 potential sage-grouse use locations as random points for every nest location. Our placement of potential sage-grouse locations was constrained to sagebrush vegetation types mapped by the inter-agency LANDFIRE project. Given that, random nest points were constrained such that they were located within 10 km of either a transmission or distribution line, and within the convex hull drawn around nest locations for each study population [39]. This established a distribution of availability that corresponded to any nesting habitat within a given study area that was also located within 10k of a power line. Using this information, we constructed our RSF using a generalized linear mixed model (GLMM) with a binomial distribution (presence vs. random) and a logit-link function implemented in the lme4 package of R. We included distance to either transmission or distribution lines as either a linear or piecewise spline covariate. Power line types were analyzed separately (i.e., two separate RSF models) to accurately measure whether a threshold response occurred to either or both power line types. Because nest site selection is also a function of percent sagebrush [13], we also tested for the additive and interactive effects of percent sagebrush cover. Lastly, we included additive effects of elevation and distance to roads as covariates to both linear and piecewise spline models (Table 1). Study area was included as a random factor.

We repeated this process for our brood site RSF, except that an additional constraint was placed on random locations. Random brood locations were constrained such that they were located: 1) within 10 km of either a transmission or distribution, 2) within the convex hull drawn around brood locations for each study population, and 3) within 10 km of each individual brood location. This established a distribution of availability that was individual specific such that random locations were in close proximity to a given brood while also meeting the prior constraints. Using this information, we constructed our RSF using a GLMM with a binomial distribution (presence vs. random) and a logit-link function implemented in the lme4 package of R. We included distance to either transmission or distribution lines as either a linear or piecewise spline covariate as described above. Although it has been suggested sage-grouse may move up in elevation as the brooding season progresses [59], we did not evaluate this in our models because it appeared to occur only in specific sites within our study area (S1 Appendix). Furthermore, the inclusion of elevation and Julian date in our models led to convergence issues, which necessitated that we focus on the effects of power lines on brood site selection. We included our measure of percent sagebrush cover as either additive or interactive effects. Lastly, we included additive effects of elevation and distance to roads to both linear and piecewise spline models (Table 1). Brood ID and study area were included as nested random effects. We built both nest and brood models using standardized coefficients (*x*_*i*_ - $\bar{x}$/s) to facilitate direct comparison within models.

### Nest and brood success

We used our nest database to construct nest models using a GLMM with a binomial distribution (success vs. failure) and a logit-link function implemented in the lme4 package of R. We included distance to either transmission or distribution lines as either a linear or piecewise spline covariate. We included our measure of percent sagebrush cover as an additive and interactive effect in the models. Lastly, we included additive effects of elevation and distance to roads to both linear and piecewise spline models (Table 1). Study area was included as a random effect in the nest analysis.

We used our brood-site database to construct nest models using a GLMM with a binomial distribution (success vs. failure) and a logit-link function implemented in the lme4 package of R. To provide a cumulative response value for this analysis, we identified the median distance to transmission and distribution lines for each brood using their associated telemetry locations. We only retained individuals in the analysis where the median value of each individual was ≤ 10 km from either a transmission or distribution line. We included distance to either transmission or distribution lines as either a linear or piecewise spline covariate. We included our measure of percent sagebrush cover as an additive and interactive effect in the models. Lastly, we included additive effects of elevation and distance to roads to both linear and piecewise spline models (Table 1). Brood ID and study area were included as nested random effects. We built both nest and brood models using standardized coefficients (*x*_*i*_ - $\bar{x}$/s) to facilitate direct comparison within models.

## Results

### Lek trend

Of the 125 leks we estimated temporal trends for, 70 leks were within 10 km of power lines (including both transmission and distribution). For these leks, a model that contained a linear effect of power lines on lek trend (ΔAICc = 0.42) was a worse fit to the data than the null model (ΔAICc = 0.00). However, support for the null model was substantially weaker (ΔAICc = 4.53) than the best-fit threshold model.

**Fig 2:**
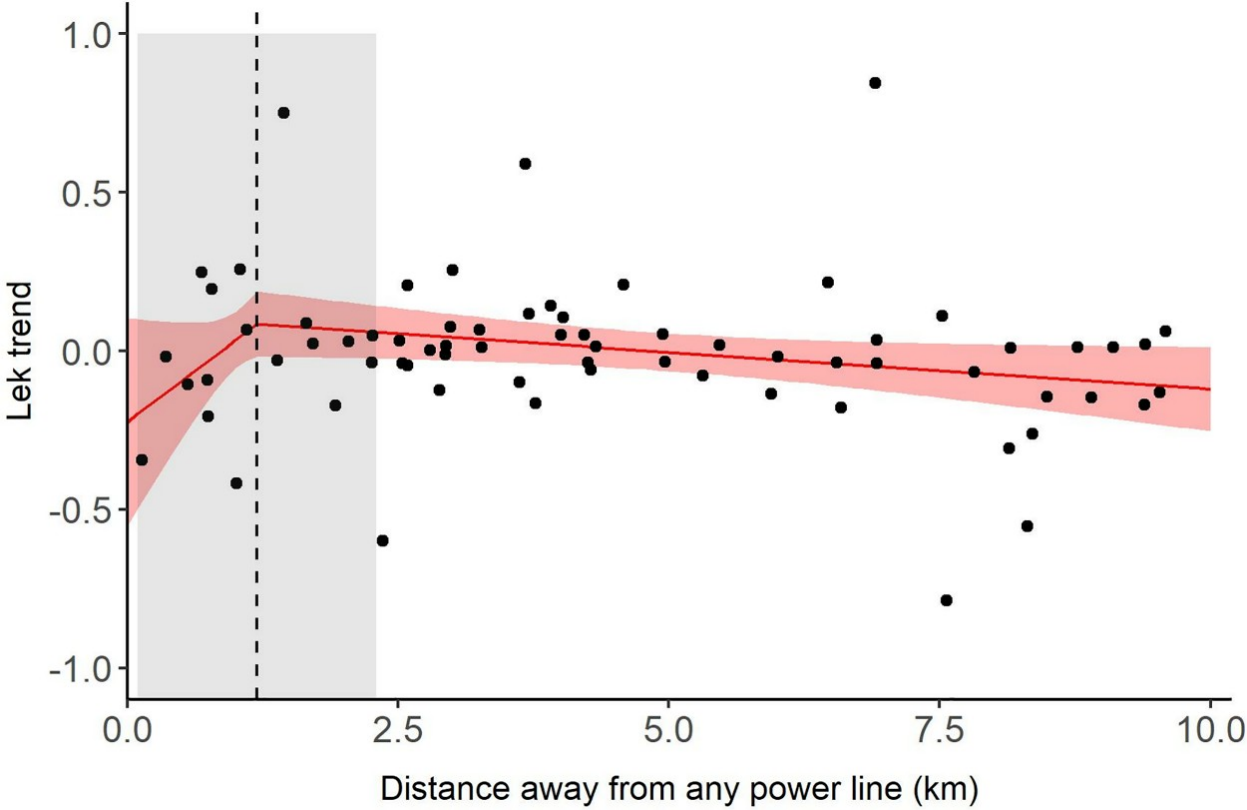
Effect of power lines on greater sage-grouse (*Centrocercus urophasianus*) lek trends and persistence in Utah, USA, 1998–2013. Lines represent the fitted values from the best-fit model of the effect of power lines on lek trends (S2 Appendix). The vertical dashed line identifies the response threshold at which sage-grouse response (i.e., lek trend) changed. Shaded areas highlight uncertainty (95% CI) around the location of the response threshold.

Thus, our data suggests that lek trends increased for leks located farther from a power line (β = 0.26, 95% CI = -0.05, 0.56) up until a distance of 1.2 (95% Threshold CI = 0.1, 2.3) km (Fig 2). After this threshold, lek trends decreased but the slope of the relationship was diminished in comparison to the pre-threshold relationship (β = -0.02, 95% CI = -0.05, -0.00).

### Lek persistence

We identified 186 leks within 10 km of transmission and distribution lines, of which 56 were considered to be persistent [55, 56]. For these leks, a model containing a linear effect of power lines on lek persistence (ΔAICc = 2.08) was a worse fit to the data than the null model. A model containing a threshold effect of distance to power lines on lek persistence did not improve model fit (ΔAICc = 2.15) suggesting that power lines did not influence lek persistence in our study (S2 Appendix).

### Nest and brood site selection

#### Nests

**Fig 3:**
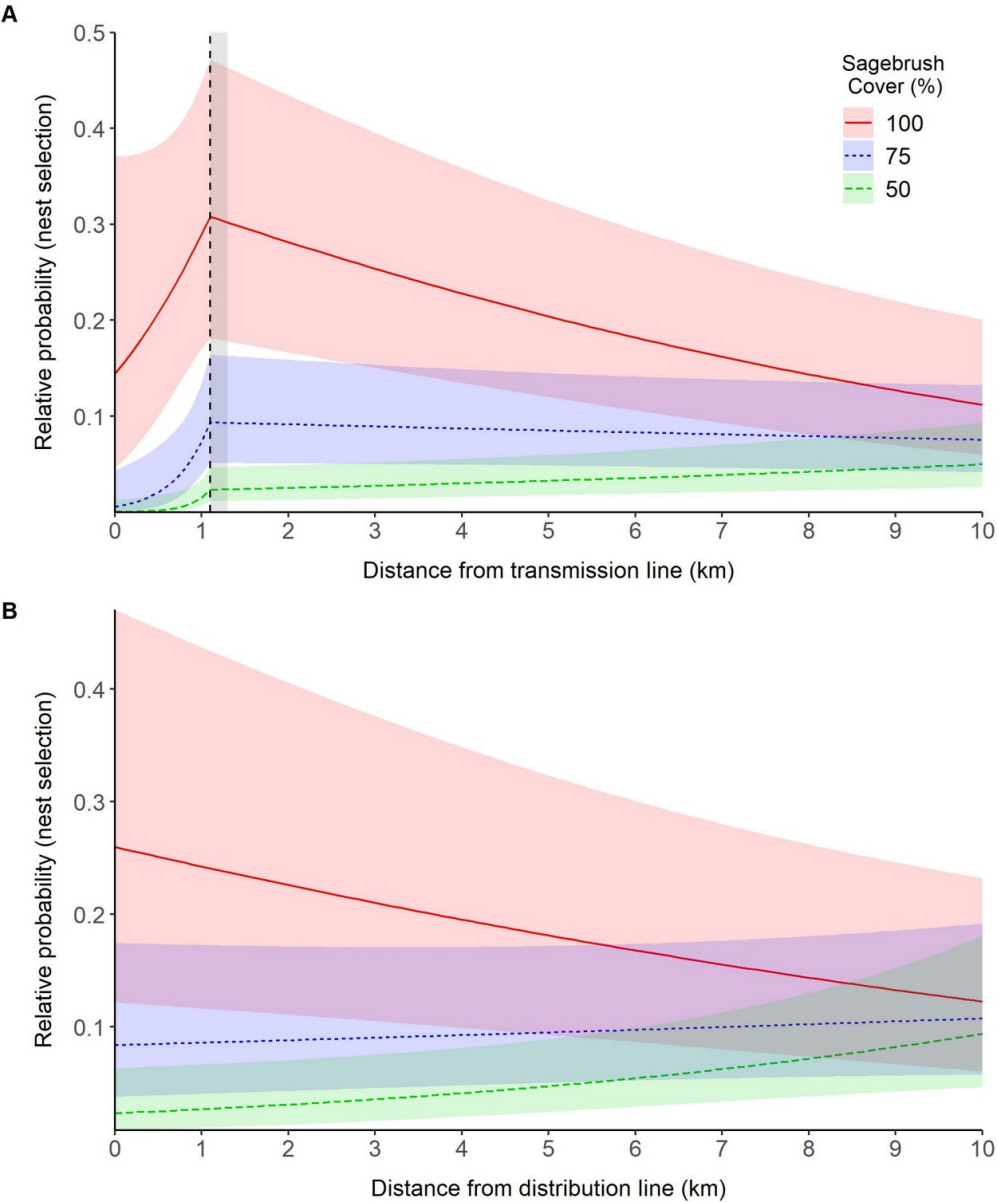
Effect of power lines on the relative probability of nest site selection of greater sage-grouse (*Centrocercus urophasianus*; sage-grouse) throughout Utah, portions of southeastern Idaho, and southwestern Wyoming, USA, 1998–2013. Lines are population-averaged fitted values from the best-fit GLMM (S3 Appendix) describing the effects of transmission lines (A) and distribution lines (B) on sage-grouse nest site selection. The vertical dashed line identifies the response threshold at which sage grouse response changes. The shaded areas highlight uncertainty (ΔAICc < 2) around the location of the response threshold.

Of 429 nests, 365 were within 10 km of a transmission line and 222 were within 10 km of a distribution line. Individual sage-grouse nests were recorded as close as 103 m and 239 m from electric power transmission and distribution lines, respectively. For these nests, a model that contained a linear effect of distance to transmission lines on the relative probability of nest-site selection was a better fit to the data than the null model (ΔAICc = 1.52). A model that contained an interaction between distance to transmission lines and percent sagebrush cover further improved model fit over the univariate linear distance to power line model (ΔAICc = 175.33). However, support for this best-fit linear model was substantially weaker (ΔAICc = 7.67) than the best fit threshold model that included an interaction between distance to transmission lines and percent sagebrush cover. Thus, our data suggests that the relative probability of nest-site selection increased as sage-grouse moved farther from transmission lines up until a distance of 1.1 (range = 1.1–1.3; S3 Appendix) km (Fig 3A). After this threshold, the relative probability of nest-site selection declined in areas of high sagebrush cover but remained relatively constant in areas of low sagebrush cover. In accordance, a linear (univariate) analysis suggested that sage-grouse selected nest sites farther from transmission lines (β = 0.10, 95% CI = -0.00, 0.21).

For nests within 10 km of a distribution line, a model that contained a linear effect of distribution lines on the relative probability of nest-site selection was a better fit to the data than the null model (ΔAICc = 0.26). A full model that included a linear effect of distance to distribution line further improved model fit over the univariate linear distance to distribution line model (ΔAICc = 63.11). The best-fit threshold model did not improve model fit (ΔAICc = 0.32) over the best-fit linear model. For our best-fit model, the relative probability of nest site selection increased as distance from distribution lines increased (β = 0.12, 95% CI = -0.08, 0.33; S3 Appendix).

#### Broods

**Fig 4:**
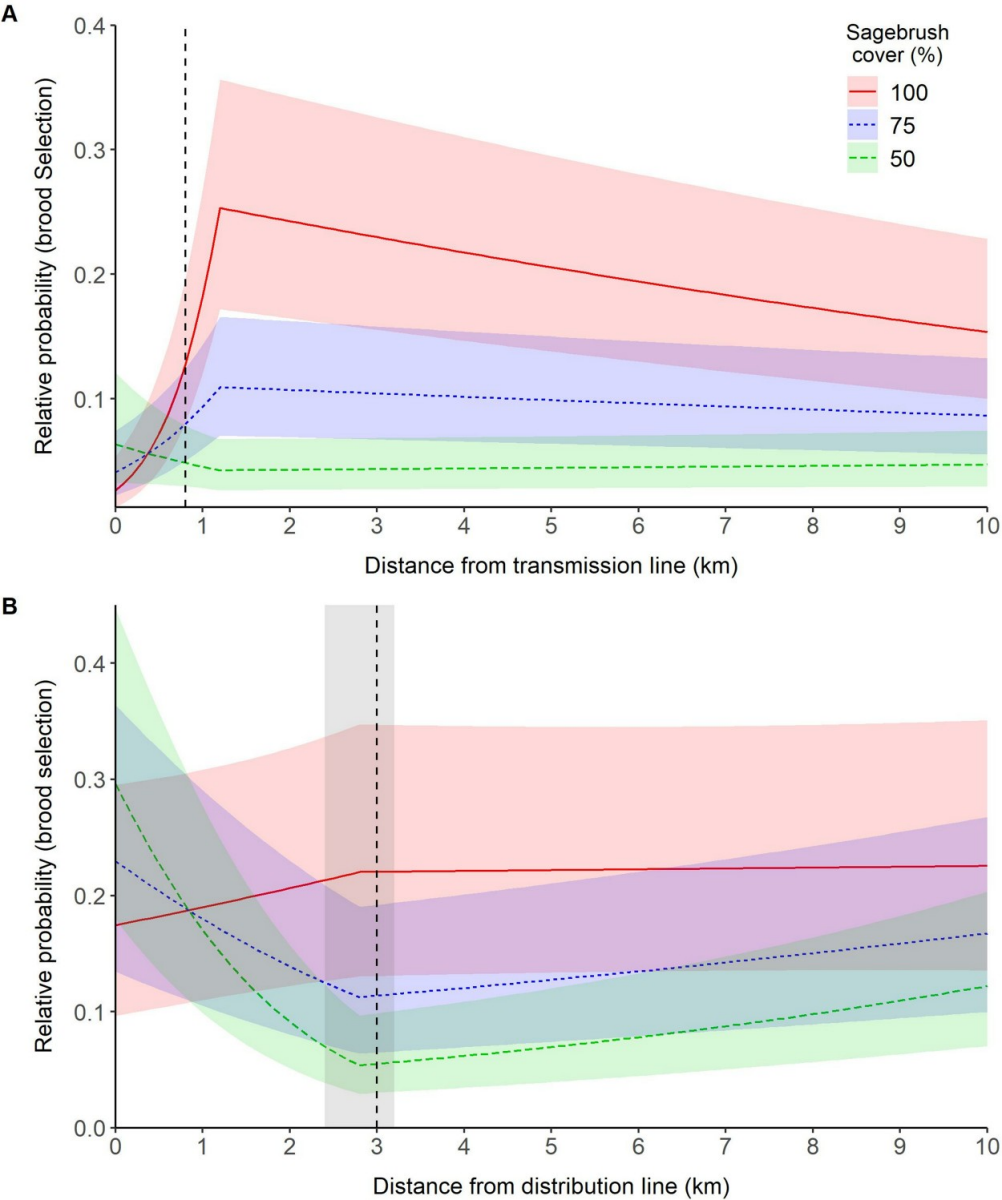
Effect of power lines on the relative probability of greater sage-grouse (*Centrocercus urophasianus*; sage-grouse) brood site selection in Utah, portions of southeastern Idaho, and southwestern Wyoming, USA, 1998–2013. Lines are population-averaged fitted values from the best-fit GLMM (S4 Appendix) describing the effects of transmission lines (A) and distribution lines (B) on sage-grouse brood site selection. The vertical dashed line identifies the response threshold at which sage grouse response changes. The shaded areas highlight uncertainty (ΔAICc < 2) around the location of the response threshold (no uncertainty was recorded for distance from transmission lines).

We recorded 3,335 brood locations during the study, of which 2,986 were within 10 km of a transmission line and 1,610 were within 10 km of a distribution line. For these broods, a model that contained a linear effect of transmission lines on brood-site selection was a better fit to the data then the null model (ΔAICc = 176.50). The full model that included a threshold effect of distance to transmission line further improved model fit over the linear distance to transmission line model (ΔAICc = 1584.34). However, support for the best-fit linear model was substantially weaker (ΔAICc = 154.13) than the best fit threshold model which included an interaction between distance to transmission lines and percent sagebrush cover. Thus, our data suggest that the relative probability of brood use increased for leks located farther from a transmission line up until a distance of 1.2 (range = 1.2–1.4; S4 Appendix) km (Fig 4). After this threshold, the relative probability of brood-site selection declined in areas of high sagebrush cover but remained relatively constant in areas of low sagebrush cover.

For brood sites within 10 km of a distribution line, a model containing a linear effect of distribution lines on the relative probability of nest-site selection was a better fit to the data than the null model (ΔAICc = 193.5). The full model that contained a linear effect of distance to distribution lines further improved model fit over the linear distance to distribution line model (ΔAICc = 638.65). However, support for the best-fit linear model was substantially weaker (ΔAICc = 126.15) than the best fit threshold model which included all covariates and interactions (S4 Appendix). Thus, our data suggests the relative probability of brood use may be positively influenced by distribution lines except in cases of high sagebrush cover until a distance of 3.0 (range = 2.4–3.2; S4 Appendix) km (Fig 4). After this threshold, the relative probability of brood-site selection remained relatively constant.

### Nest and brood success

#### Nests

**Fig 5:**
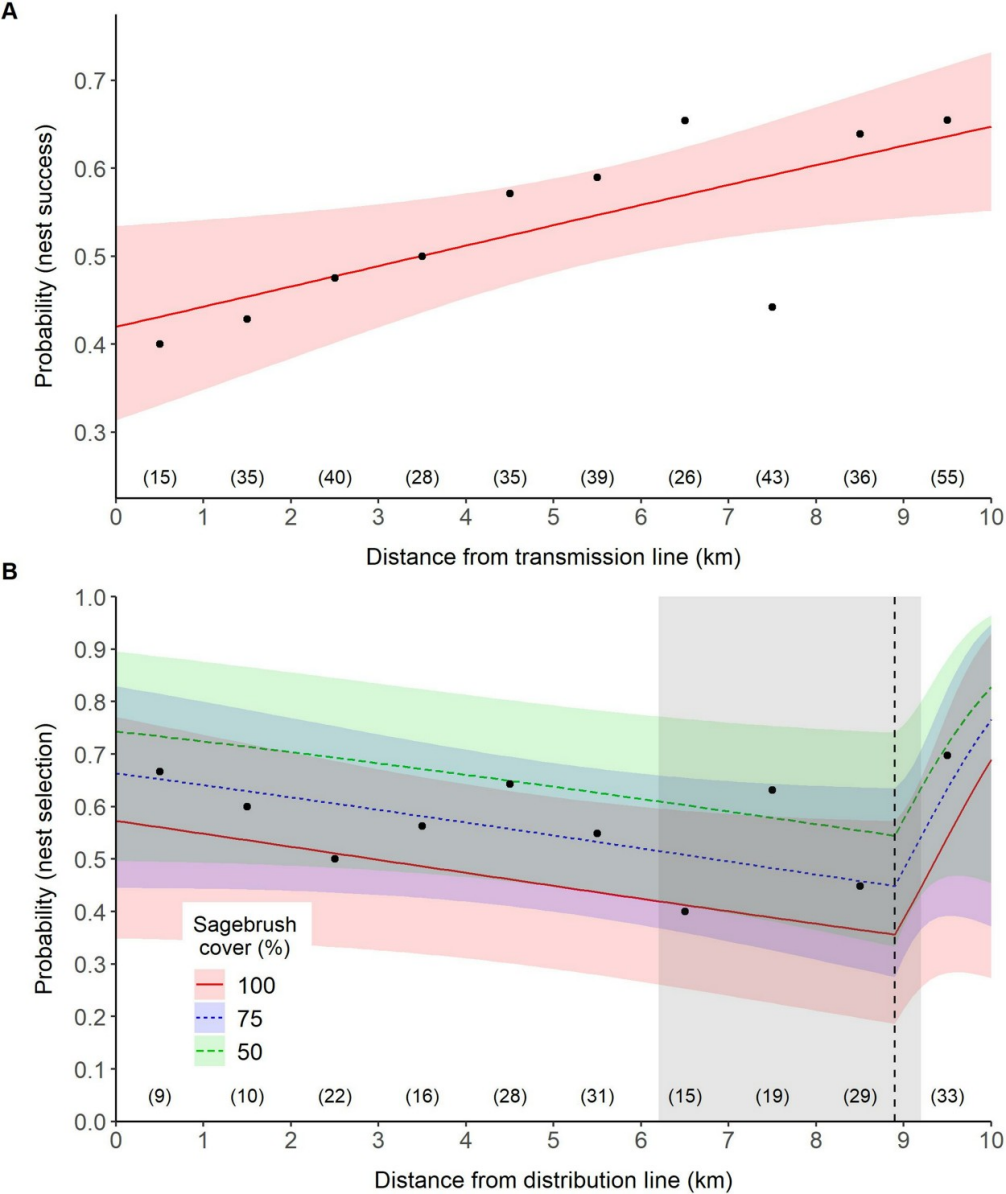
Effect of power lines on the greater sage-grouse (*Centrocercus urophasianus*; sage-grouse) in Utah, portions of southeastern Idaho, and southwestern Wyoming, USA, 1998–2013. Lines are population-averaged fitted values from the best-fit GLMM (S5 Appendix) describing the effects of transmission lines (A) and distribution lines (B) on sage-grouse nest success. The vertical dashed line identifies the response threshold at which sage-grouse response changes. The shaded areas highlight uncertainty (ΔAICc < 2) around the location of the response threshold. Circles represent a binning of data points that informed the model; sample sizes within each 1 km bin are identified in parentheses. For example, 15 nests were recorded between 0–1 km from a transmission line, of which 40% (n = 6) persisted.

We recorded 422 nests during the study, of which 83% (n = 352) were within 10 km of a transmission line and 50% (n = 212) were within 10 km of a distribution line. For these nests, a model that contained a linear effect of transmission lines on nest success was a better fit to the data than the null model (ΔAICc = 2.95). A model that include the linear effect of distance to transmission lines and distance to roads improved model fit over the univariate linear distance to transmission model (ΔAICc = 1.00). The best-fit threshold model did not improve model fit (ΔAICc = 0.94) over the best-fit linear model. For our best-fit model, the relative probability of nest success increased as distance from transmission lines increased (β = 0.27, 95% CI = 0.05, 0.48; S3 Appendix; Fig 5).

For nests within 10 km of a distribution line, a model containing a linear effect of distribution lines on nest success did not improve model fit (ΔAICc = 1.77) over the null model. However, support for the null model was weaker (ΔAICc = 1.00) than the best-fit threshold model which included an additive effect of percent sagebrush cover. Thus, our data suggested that the probability of nest success declined as you moved farther from a distribution line (β = -0.27, 95% CI = -0.64, 0.08; Fig 5) up until a distance of 8.9 (range = 6.2–9.2; S5 Appendix) km, after which, nest success increased (β = 3.45, 95% CI = -1.03, 8.40). It is likely that the decrease in nest success beyond the threshold was driven by our sample size (n = 35 / 222) beyond 8.9 km. Of those 35 nests, 77% (n = 27) were located in two study areas which had a combined 66% nest success suggesting that these areas influenced our model results beyond 8.9 km.

#### Broods

We recorded 434 broods during the study, of which 89% (n = 386) were within 10 km of a transmission line and 47% (n = 206) were within 10 km of a distribution line. Brood locations were documented as close as 205 m and 24 m from transmission and distribution power lines, respectively. Because our data set contained multiple locations for each brood that were recorded regularly throughout the season, we were also able to determine if some broods may have traveled under power lines. We recorded eight radio-marked female sage-grouse with broods (4%) crossing under transmission lines 27 times. Eleven brooding females (8%) crossed under distribution lines 86 times. For broods that crossed distribution lines, the median number of crossings per brood was three (range 2–33). Fifteen broods (6%) crossed under both transmission and distribution lines.

**Fig 6:**
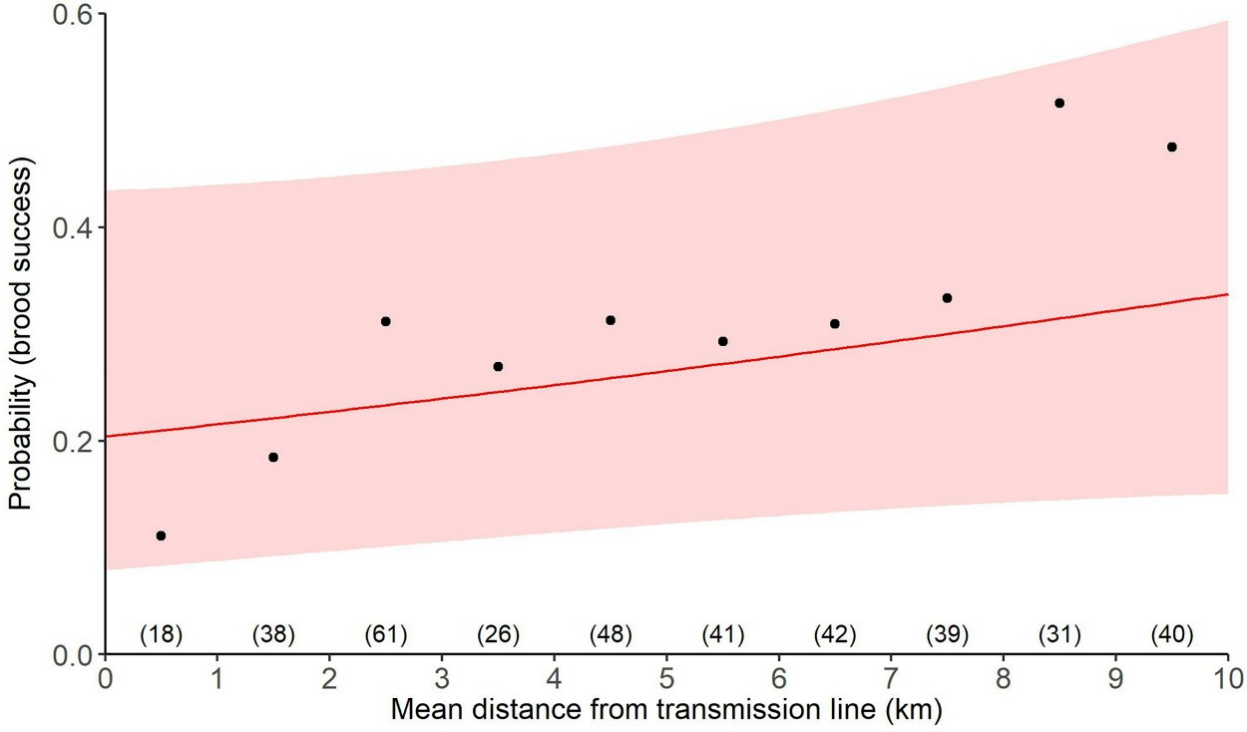
Effect of power lines on the greater sage-grouse (*Centrocercus urophasianus*; sage-grouse) in Utah, portions of southeastern Idaho, and southwestern Wyoming, USA, 1998–2013. Lines are population-averaged fitted values from the best-fit GLMM (S6 Appendix) describing the effects of transmission lines on sage-grouse brood success. Circles represent a binning of data points that informed the model; sample sizes within each 1 km bin are identified in parentheses. For example, 18 nests were recorded between 0–1 km from a transmission line, of which 11% (n = 2) persisted.

For these broods, a model that contained a linear effect of transmission lines on brood success was a better fit to the data than the null model (ΔAICc = 1.13). A model that included a linear effect of transmission line and elevation farther improved model fit over the univariate linear distance to transmission line model (ΔAICc = 0.70). The best-fit threshold model did not improve model fit (ΔAICc = 0.52) over the best-fit linear model. For our best-fit model, the relative probability of brood success increased as distance from transmission lines increased (β = 0.19, 95% CI = -0.09, 0.47; S3 Appendix; Fig 6).

For broods within 10 km of a distribution line, a model that contained a linear effect of distribution on brood success was a worse fit than the null model (ΔAICc = 1.72). A model that contained a threshold effect of distance to distribution lines on brood success did not improve model fit (ΔAICc = 1.83) beyond the null model (S6 Appendix).

### Preponderance of evidence

There was support (< ΔAICc of 2.0) for a nonlinear model being our best-fitting model for all analyses except lek persistence (Table 2). For lek trend, our best-fit model suggested that there was a negative effect of power lines up until a distance of 1.2 km. We report similar results for nest-site selection (1.1 km), nest success (2.4 km), and brood success (1.3 km) relative to transmission lines (positive coefficient for D1 in Table 2). We observed the opposite pattern (higher selection for distances close to transmission lines) for brood-site selection, however, these main coefficient effects were influenced by a percent sagebrush cover interaction which suggested a negative effect of transmission lines (Fig 4).

**Table 2:** Preponderance of evidence Table. Delta AIC scores corrected for small sample sizes for the best-fit null, linear, and nonlinear model for each analyses.

Analyses		Null Model ΔAICc	Best Linear Model ΔAICc	Best Nonlinear Model ΔAICc	Threshold (km)	Best Nonlinear Model Dist1	Best Nonlinear Model Dist2
							
Lek analyses							
	Lek trend	0.47	0.88	0.00	1.2	0.26	-0.02
	Lek persistence	0.00					
							
Transmission line analyses							
	Nest selection (transmission line)			0.00	1.1	9.73	0.10
	Brood selection (transmission line)			0.00	1.2	-0.01	0.00
	Nest success (transmission line)		0.00	0.94	2.4	1.09	0.18
	Brood success (transmission line)	1.83	0.00	0.52	1.3	4.60	0.14
							
Distribution line analyses							
	Nest selection (distribution line)		0.00	0.32	9.5	0.19	-6.74
	Brood selection (distribution line)			0.00	3.0	-1.41	0.26
	Nest success (transmission line)		0.00	0.94	2.4	1.09	0.18
	Brood success (distribution line)	0.00	1.72	1.83	6.7	-0.38	0.88

Best-fit models were only displayed if they were within < ΔAICc of 2.0. For the best-fit nonlinear model, we identified the threshold and the main effect marginal coefficient before (D1) and after (D2) the threshold. Across all analyses, our data suggests a negative effect of transmission lines up to 2.4 km and no effect of distribution lines.

In comparison, our data suggested that sage-grouse may be negatively affected by distribution lines during the nesting period (positive coefficient for D1 in Table 2) but positively selecting for areas near distribution lines during the brood period (positive coefficient for D1 in Table 2). It is also important to note that our main effect coefficients for nest-site selection were influence by a percent sagebrush cover interaction that suggests that distribution lines may have a positive effect of nest-site selection (Fig 3B). As such, our overall weight of evidence approach suggests there was a negative effect of transmission lines up until a threshold of 2.4 km and there was general lack of support for a distribution line effect.

## Discussion

Our results suggested that different types of power lines can have varying effects on sage-grouse demographics, and percent sagebrush cover may influence these effects. For example, lek trends were negatively affected by power lines up until a distance of 1.2 (95% CI = 0.1–2.3) km. When we were able to separate sage-grouse responses according to power line type, transmission lines appeared to have greater impacts to sage-grouse than distribution lines. Thus, our analyses yielded a data-driven 2.3-km BMP buffer zone around leks for the construction of new transmission power lines in occupied sage-grouse habitats. However, sage-grouse nest and brood locations where still documented within this BMP buffer zone. Because distribution lines had less of an impact on sage-grouse demographics than transmission lines, we recommend site specific planning as appropriate for distribution lines, which may include buffers, micrositing, habitat reclamation, other BMPs, or a combination of these practices.

Based on our analysis, sage-grouse avoided nesting within 1.1 km of transmission lines and nest success was most negatively affected at distances up until 2.4 km (Table 2). We did not detect an effect of distance to distribution lines on sage-grouse nest-site selection. Similarly, brooding female sage-grouse tended to avoid the immediate proximity of transmission lines up to a distance of 1.2 km, whereas they did not avoid distribution lines. Similar to our site-selection analyses, transmission lines had a negative effect on sage-grouse nest success up to a distance of 2.4 km, whereas, sage-grouse nests located closer to distribution lines were more successful. Transmission lines also had a negative effect on brood success up to a distance of 1.3 km, whereas distribution lines had no effect.

These preponderance of evidence suggests that sage-grouse may be more impacted by transmission lines than to distribution lines. It remains unclear, however, whether this was driven by habitat characteristics. In our study area, transmission lines were located at lower elevations on the landscape and farther from developed areas. In contrast, distribution lines were located in areas with greater anthropogenic development and habitat fragmentation than transmission lines, as they directly provide electrical power to homes, communities, businesses, and industry [60]. Thus, habitat availability alone may help explain increased avoidance of transmission lines but not distribution lines (i.e., sage-grouse could not avoid distribution lines without encountering other anthropogenic structures, whereas sage-grouse could have ranged in larger areas surrounding transmission lines in otherwise intact habitat). This is likely in our study areas because sage-grouse seasonal movements in Utah reflect habitat availability [39]. It may also be possible that the interspersed, and thus, more homogenous spatial arrangement of distribution lines on the landscape may have facilitated habituation by sage-grouse [34].

The high density and proximity of secondary roads adjacent to power lines may also explain sage-grouse avoidance of power lines [11, 22, 32]. In our study area, we observed both selection for (brood-site selection-distribution; S4 Appendix, nest-success: transmission; S5 Appendix) and avoidance of roads (brood-site selection-transmission; S4 Appendix). This inconclusive effect of roads may be in part due to the correlation of power lines and roads. In areas where transmission lines and distribution lines had been constructed, 54% and 69% of sage-grouse locations, respectively, were within 100 m of a road (S7 Appendix). These roads have established linear corridors that may exceed 300 m or more and may include unimproved power line access roads, county roads, and/or state/interstate highways [11]. Thus, sagebrush habitats located < 1 km of the power lines may have been degraded by historic road and power line construction that contributed to sage-grouse habitat loss and therefore site avoidance. Similar to other studies, [23], we were unable to separate these effects (anthropogenic development from and tall structures) due to small sample size. Moreover, Johnson et al. [34] and Wisdom et al. [32] cautioned that retrospective studies may be biased in that many of the factors affecting sage-grouse ecology were in place prior to their studies [11]. Despite this, our study does represent the first comprehensive examination of the direct and indirect effects of power lines on sage-grouse at a landscape scale. This is important because published research on the effects of these structures on the reproductive fitness of any grouse species is lacking [3].

Alternatively, it has been hypothesized that the avoidance of transmission lines is due to direct predation facilitated by increased predator visibility and increased raptor and corvid nesting substrates [11, 28, 30, 31]. Scientific evidence is still lacking as to whether sage-grouse instinctively avoid power lines specifically to avoid predators [11, 37], but given that transmission lines were 8–30 meters taller than distribution lines in our study area, this may be a potential mechanism to explain sage-grouse avoidance. It is also important to note that Lammers and Collopy [61] and Prather and Messmer [62] both documented high use of electric power distribution lines as perch sites for raptors and corvids in or near our study areas which suggest that predation risk may be higher closer to power lines. Our results that demonstrated increased avoidance of transmission lines and lower survival in areas near them corroborates this. However, our study did document sage-grouse movements beneath power lines suggesting predation may not be as influential as sometimes stated. As such, this debate remains unresolved because of the difficulty in connecting predation risks to various combinations or types of power lines and avian predator species [11, 23, 37].

Although we were unable to identify the mechanism responsible for sage-grouse avoidance of power lines, we were able to provide the first landscape-scale empirical evaluation of the effects of power lines on sage-grouse during the breeding season. Our evaluation supports the broad-scale analysis by Wisdom et al. [32] and Gibson, Blomberg (30) that distance to transmission lines had a potential negative influence on lek persistence. However, our analyses extended beyond Wisdom et al. [26] in that we identified a BMP buffer zone of 2.3 km from active leks for power lines that could avoid and minimize the potential effects of new power line construction on sage-grouse ecology.

For comparison, a 3.0 km minimum BMP buffer zone has been previously recommended to mitigate the effects of tall structures on seasonal sage-grouse habitat [13, 63–65]. This BMP buffer zone was recommended because available range wide nesting data suggested most sage-grouse nest within 3.2 km of known leks [66]. In our study areas, nest success increased as females selected nesting sites farther from transmission lines. This effect was greatest up to a distance of 2.4 km. However, long-term sage-grouse nest location data collected in Utah and other states suggested that some female sage-grouse may nest up to 5.0 km or more from known leks [39, 67]. This may explain why nesting success increased, but at a slower rate, beyond the 2.4 km threshold (Table 2).

The BLM National Technical Team (NTT) reviewed data regarding distances from nest locations to the nearest leks where female sage-grouse were captured [68]. Based on these data, the BMP buffer zone would need to exceed a 6.0 km radius around leks to protect most of the nesting female sage-grouse. However, the NTT acknowledged a 6.0 km buffer would not be practical because of oil and gas leasing requirements and that within priority habitats existing lek-based BMP buffers may overlap precluding development [68]. As such, our results lend support to Connelly et al. [13], the Wyoming Sage-grouse Executive Order [69], and APLIC [36] recommendations of placing new electric transmission lines within existing corridors. If this is unfeasible, we suggest a 2.3 km buffer for the construction of transmission lines be applied around active leks to avoid and minimize impacts to nesting habitat in Utah. It is unclear from our data whether an active lek buffer associated with distribution lines is required around nesting habitat, thus, we would recommend that new distribution lines be co-located with existing disturbance if possible.

Beck et al. [70] estimated that 29,821 km^2^ (13.6%) of Utah provided sage-grouse habitat. Connelly et al. [71] and Knick et al. [24] suggested that electric power transmission lines could have an impact on 40–50% (11,928–14,911 km^2^) of all sagebrush within designated sage-grouse management areas. However, we found that only 10% of transmission lines and 7% of distribution lines within our study areas were within 10 km of occupied sage-grouse habitat suggesting that power lines may be less influential to overall sage-grouse habitat than previously thought in Utah. Moreover, if we applied a 2.3 km buffer zone around all known electric power transmission (1,698 km) and distribution (1,496 km) lines, our analysis suggests that power lines may only have an ecological effect on 22.6% (6,817km^2^) of available sage-grouse breeding habitat in our study areas.

Our results highlighted the importance of maintaining suitable sagebrush cover in areas where power lines occur on the landscape, as percent sagebrush cover increased grouse selection of sites for nesting and brooding increased despite the presence of power lines. In our study areas, percent sagebrush cover improved as distance to transmission lines increased (β = 3.28, *p* ≤ 0.01). This may explain why nest and brood success continued to increase as sage-grouse females moved farther from transmission lines. Our results validate the value of reclaiming sagebrush habitats in areas in close proximity to power lines.

Female sage-grouse demonstrated high selection preferences for sites within ~ 1.1 km of transmission lines during the nesting and brooding period, which suggests these sites provide additional benefits which we did not account for in our models. In such cases, management strategies that increased the availability of useable sagebrush habitat space (i.e., removal of conifer encroachment and vegetation management to encourage low growing species) in areas where power lines occur could enhance population stability [39, 72, 73] and potentially counteract the negative impacts of power lines. However, it is possible that habitat improvement strategies could lead to population sink dynamics if sage-grouse are attracted to power lines and subsequently experience negative population growth due to factors such as increased predation [30]. Thus, managers should evaluate this practice on a case-by-case basis.

Currently 87% of the sage-grouse range is grazed by livestock [24]. Dahlgren et al. [54, 73] reported increased sage-grouse production on working landscapes grazed by domestic livestock using rotational grazing practices. Utility ROW vegetation management practices may serve a similar function to livestock grazing, as both reduce tall growing species and encourage growth of grassland and shrub species. Sandford et al. [72] reported increased sage-grouse nest survival and brood success in sagebrush landscapes where conifer encroachment was reduced. Messmer [74] suggested implementation and evaluation of these landscape-level management practices should be considered as part of the integrated toolkit to mitigate the potential effects anthropogenic activities have on sage-grouse and their habitats.

Our study represents the first landscape-level comparison within a state’s jurisdictional boundary that examined the relationship between power line classification and known sage-grouse nest and brood locations. Despite our comprehensive approach, we were still unable to resolve a number of concerns that may influence sage-grouse responses to power lines due in a large part to our limited samples sizes. Specifically, our sample size limited our ability to evaluate how structure height and design may differentially influence sage-grouse response beyond our general classifications (e.g., distribution vs transmission). Similarly, it is unclear whether structure design may play a role in sage-grouse response given the variation in available perching and nesting substrates that power lines provide to avian predators [11]. We were also unable to evaluate the extent to which placement of new power lines within anthropogenic corridors (e.g., power lines, transportation) reduces the overall negative influence of sage-grouse (e.g., [29]), and whether those benefits to sage-grouse are capped at some development threshold (e.g., 2 vs. 4 power lines in a corridor). Due to the propensity of roads in proximity to power lines throughout our study area, we were also unable to distinguish between potential independent impacts from roads versus power lines. Lastly, we documented increased use of elevation during the brood period, however we were unable to explicitly evaluate how seasonal movement behaviors may interact with sage-grouse response to power lines (distance to power lines x elevation interaction), as birds may move away from power lines in response to other unrelated habitat factors. Given these limitations, we encourage future research that addresses these challenges. The increasing use of GPS technology in wildlife studies, including sage-grouse, and the larger sample sizes they provide, may provide new opportunities to evaluate these unanswered questions.

## Conclusions

Our research is the first to quantify appropriate BMP buffer distances based on known sage-grouse seasonal habitat-use data for the construction of new electric power transmission and distribution lines in sage-grouse habitats. Our research also demonstrated that interactions between percent sagebrush cover and type of power line affect sage-grouse responses. Based on our results, we recommend a hierarchical approach for avoiding and minimizing the potential impacts of power lines on sage-grouse. New transmission power lines should be placed in existing utility or transportation corridors where feasible. If co-location is not feasible, lek buffers of 2.3 km for new transmission lines should be used to mitigate the potential impacts to sage-grouse. It is questionable from our results, however, that this buffer would also apply to distribution lines given the uncertainty we observed regarding sage-grouse response to distribution lines in our habitat selection and reproductive success analyses. In addition, other regulatory or resource conflicts (i.e., electric utilities are required by law to provide service to requesting customers and to minimize impacts to other resources–such as wetlands, cultural or paleontological resources, etc.) can result in new construction within a sage-grouse buffer in some circumstances. Thus, we recommend managers continue to incorporate habitat protection and reclamation as conservation strategies for new power line construction, and population monitoring (i.e., lek counts) as feedback mechanisms to evaluate the effectiveness of these strategies. The use of applicable BMPs, such as the sage-grouse BMPs for electric utilities developed by APLIC, should be considered and implemented on a project-by-project basis. It is important that wildlife resource agencies, land management agencies, electric utilities, and private landowners work together to address site-specific aspects of new power line locations and sage-grouse habitats. More broadly, the methodological process employed here can be used widely to evaluate the influence of anthropogenic structures on grouse species globally. With this information, land managers will be better suited to minimize impacts from ongoing human infrastructure construction on wildlife.

## Supporting information

S1 Appendix (DOCX)

S2 Appendix (DOCX)

S3 Appendix (DOCX)

S4 Appendix (DOCX)

S5 Appendix (DOCX)

S6 Appendix (DOCX)

S7 Appendix (DOCX)

## References

[1] Goldenwijk KK, Beusen A, van Drecht G, de Vos M. The HYDE 3.1 spatially explicit database of human induced global land-use change over the past 12,000 years. Global Ecol Biogeogr. 2010; 20: 73–86.

[2] Benítez-López A, Alkemade R, Verweij PA. The impacts of roads and other infrastructure on mammal and bird populations: a meta-analysis. Biol Conserv. 2010; 143: 1307–1316.

[3] Hovick TJ, Elmore RD, Dahlgren DK, Fuhlendorf SD, Engle DM. Review: evidence of negative effects of anthropogenic structures on wildlife: a review of grouse survival and behaviour. J Appl Ecol. 2014; 51: 1680–1689.

[4] Hansen AJ, Knight RL, Marzluff JM, Powell S, Brown K, Gude PH, et al Effects of exurban development on biodiversity: patterns, mechanisms, and research needs. Ecol Appl. 2010; 15; 1893–1905.

[5] Kuvlesky WP Jr, Brennan LA, Morrison ML, Boydston KK, Ballard BM, Bryant FC. Wind energy development and wildlife conservation: challenges and opportunities. J Wildlife Manage. 2007; 71: 2487–2498.

[6] Leu M, Hanser SE, Knick ST. The human footprint in the west: a large-scale analysis of anthropogenic impacts. Ecol Appl. 2008; 18: 1119–1139. 18686576 10.1890/07-0480.1

[7] Bevanger K. Biological and conservation aspects of bird mortality caused by electricity power lines: a review. Biol Conserv. 1998; 86: 67–76.

[8] Bui TD, Marzluff JM, Bedrosian B. Common raven activity in relation to land use in western Wyoming: implications for greater sage-grouse reproductive success. Condor 2010; 112: 65–78.

[9] Wilcove DS, Wikelski M. Going, going, gone: is animal migration disappearing. Plos Biol. 2010 6(7): e188 10.1371/journal.pbio.0060188 PMC2486312 18666834

[10] Smith JA, Dwyer JF. Avian interactions with renewable energy infrastructure: an update. Condor. 2016; 118: 411–423.

[11] Messmer TA, Hasenyager R, Burruss J, Liguori S. Stakeholder contemporary knowledge needs regarding the potential effects of tall structures on sage-grouse. Human Wildlife Interactions. 2013; 7: 273–298.

[12] Mabey S, Paul E. Critical literature review: impact of wind energy and related human activities on grassland and shrub-steppe birds. National Wind Coordinating Collaborative. 2007; Available from: https://nationalwind.org/wp-content/uploads/2013/05/Critical-Lit-Review-Oct-2007.pdf

[13] Connelly JW, Schroeder MA, Sands AR, Braun CE. Guidelines to manage sage-grouse populations and their habitat. Wildlife Soc Bull. 2000; 28: 967–985.

[14] Connelly JW, Braun CE. Long-term changes in sage-grouse *Centrocercus urophasianus* populations in western North America. Wildlife Biol. 1997; 3/4: 123–128

[15] U.S. Fish and Wildlife Service (USFWS). Greater Sage-grouse (*Centrocercus urophasianus*) Conservation Objectives: Final Report. U.S. Fish and Wildlife Service, Denver, CO. February 2013.

[16] U.S. Fish and Wildlife Service (USFWS). Notice of 12-Month Petition Findings for Petitions to List Greater Sage-grouse as Threatened or Endangered. 2010; Available from (http://www.regulations.gov and www.fws.gov).

[17] Walker BL, Naugle DE, Doherty KE. Greater sage-grouse population response to energy development and habitat loss. J Wildlife Manage. 2007; 71: 2644–2654.

[18] Holloran MJ, Kaiser RC, Hubert WA. Yearling greater sage-grouse response to energy development in Wyoming. J Wildlife Manage. 2010; 74: 65–72.

[19] Harju SM, Dzialak MR, Taylor RC, Hayden-Wing LD, Winstead JB. Thresholds and time lags in effects of energy development on greater sage-grouse populations. J Wildlife Manage. 2010; 74: 437–448.

[20] Lyon AG, Anderson SH. Potential gas development impacts on sage grouse nest initiation and movement. Wildlife Soc Bull. 2003; 31: 486–491.

[21] Doherty KE, Naugle DE, Walker BL, Graham JM. Greater sage-grouse winter habitat selection and energy development. Journal of Wildlife Management. 2008; 72: 187–195.

[22] Utah Wildlife in Need Foundation (UWIN). Contemporary knowledge and research needs regarding the potential effects of tall Structures on sage-grouse (*Centrocercus urophasianus* and *C. mimimus*). 2010; Available from https://utahcbcp.org/ou-files/TallStructuresReportSeptember202010.pdf.

[23] Walters K, Kosciuch K, Jones J. Can the effect of tall structures on birds be isolated from other aspects of development? Wildlife Soc Bull. 2014; 38: 250–256.

[24] Knick ST, Hanser SE, Miller RF, Pyke DA, Wisdom MJ, Finn SP, et al Ecological influence and pathways of land use in sagebrush Chapter 13 in Studies in Avian Biology. No. 38. Berkeley: University of California Press; 2011.

[25] Marzluff JM, Knick ST, Vekasy MS, Schueck LS, Zarriello TJ. Spatial use and habitat selection of golden eagles in southwestern Idaho. Auk. 1997; 114: 673–687.

[26] McIntyre CL. Patterns in nesting area occupancy and reproductive success of golden eagles (*Aquila chrysaetos*) in Denali National Park and Preserve, Alaska, 1988–99. J Raptor Res. 2002; 36: 50–54

[27] Boarman WI, Heinrich B. Common raven (*Corvus corax*) In: Poole A, Gill F, editors. The Birds of North America, No. 476. Philadelphia: The Birds of North America, Inc.; 1999 Pp. 1–31.

[28] Dinkins JB, Conover MR, Kirol CP, Beck JL, Frey SN. Greater sage-grouse (*Centrocercus urophasianus*) select habitat based on avian predators, landscape composition, and anthropogenic features. Condor; 2014; 116: 629–642.

[29] Hansen EP, Stewart AC, Frey SN. Influence of transmission line construction on winter sage-grouse habitat use in Southern Utah. Human Wildlife Interactions. 2016; 10: 169–187.

[30] Gibson D, Blomberg EJ, Atamian MT, Espinosa SP, Sedinger JS. Effects of power lines on habitat use and demography of greater sage-grouse (*Centrocercus urophasianus*). Wild Monog. 2014; 92: 319–330

[31] Dinkins JB, Conover MR, Kirol CP, Beck JL, Frey SN. Greater sage-grouse (*Centrocercus urophasianus*) hen survival: effects of raptors, anthropogenic and landscape features, and hen behavior. Can J Zoo. 2014; 92: 319–30.

[32] Wisdom MJ, Meinke CW, Knick ST, Schroeder MA. Factors Associated with extirpation of sage-grouse Chapter 19 in Studies in Avian Biology No. 38. Berkeley: University of California Press; 2011.

[33] Westover M, Baxter J, Baxter R, Day C, Jensen R, Petersen S, et al Assessing greater sage-grouse selection of brood-rearing habitat using remotely-sensed imagery: can readily available high-resolution imagery be used to identify brood-rearing habitat across a broad landscape? PloS one. 2016; 11:e0156290 10.1371/journal.pone.0156290 27218829 PMC4878777

[34] Johnson DJ, Holloran MJ, Connelly JW, Hanser SE, Amundson CL, Knick ST. Influences of environmental and anthropogenic features on greater sage-grouse populations, 1997–2007 Chapter 17 in Studies in Avian Biology No. 38. Berkeley: University of California Press; 2011.

[35] U.S. Fish and Wildlife Service (USFWS). Interim guidelines to avoid and minimize wildlife impacts from wind turbines. 2003; Available from: (http//www.fws.gov/r9dhcbfa/windenergy.htm).

[36] Avian Power Line Interaction Committee (APLIC). Best Management Practices for Electric Utilities in Sage-Grouse Habitat. Washington, DC: Edison Electric Institute and APLIC; 2015.

[37] Manier DJ, Bowen ZH, Brooks ML, Casazza ML, Coates PS, Deibert PA, et al Conservation buffer distance estimates for Greater Sage-grouse: a review. Geophysical J Intern. 2014; 200: 200–215.

[38] Steenhof K, Kochert MN, Roppe JA. Nesting by raptors and common ravens on electrical transmission line towers. J Wildlife Manage. 1993; 57: 271–281.

[39] Dahlgren DK, Messmer TA, Crabb BA, Larsen RT, Black TA, Frey SN, et al Seasonal Movements of Greater Sage-Grouse Populations in Utah: Implications for Species Conservation. Wildlife Soc Bull. 2016a; 40: 288–299

[40] Stiver, SJ, Apa AD, Bohne JR, Bunnell SD, Deibert PA, Gardner SC, et al. Greater Sage-grouse Comprehensive Conservation Strategy. 2006. Western Association of Fish and Wildlife Agencies. Unpublished Report. Cheyenne, Wyoming.

[41] State of Utah. Final Conservation plan for greater sage-grouse in Utah. February 14, 2013.

[42] West NE. Great Basin-Colorado Plateau sagebrush semi-desert In West NE, editor. Temperate deserts and semi-deserts. Amsterdam: Elsevier; 1983 pp. 331–349.

[43] Connelly JW, Reese KP, Schroeder MA. Monitoring of greater sage-grouse habitats and populations Station Bulletin 80. Moscow: College of Natural Resources Experiment Station, University of Idaho; 2003.

[44] Wallestad R, Pyrah D. Movement and nesting of sage grouse hens in central Montana. J Wildlife Manage. 38: 630–633.

[45] Gregg MA, Crawford JA, Drut MS, DeLong AK. Vegetational cover and predation of sage grouse nests in Oregon. J Wildlife Manage. 1994; 58: 162–166.

[46] Baddeley A, Rubak E, Turner R. Spatial Point Patterns: Methodology and Applications with R London: Chapman and Hall/CRC Press; 2015.

[47] R Core Development Team. R: A language and environment for statistical computing. R Foundation for Statistical Computing, Vienna, Austria. 2014; Available from: http://www.R-project.org/.

[48] US Census Bureau. TIGER Products. Washington DC, USA; Available from https://www.census.gov/geo/maps-data/data/tiger.html

[49] Burnham KP, Anderson DR. Model selection and multi-model inference: a practical information-theoretic approach. 2nd ed New York: Springer-Verlag; 2002.

[50] MacNulty DR, Tallian A, Stahler DR, Smith DW. Influence of group size on the success of wolves hunting bison. PloS one. 2014; 9(11), e112884 10.1371/journal.pone.0112884 25389760 PMC4229308

[51] Arnold TW. Uninformative parameters and model selection using Akaike’s Information Criterion. J Wildlife Manage. 2010; 74: 1175–1178.

[52] Anderson DR, Burnham KP, Thompson WL. Null hypothesis testing: problems, prevalence, and an alternative. J Wildlife Manage. 2000; 64: 912–923.

[53] Burnham KP, Anderson DR. P values are only an index to evidence: 20th–vs. 21st–century statistical evidence. Ecology 2014; 95: 627–630. 24804444 10.1890/13-1066.1

[54] Dahlgren DK, Larsen RT, Danvir R, Wilson G, Thacker ET, Black TA, et al Greater sage-grouse and range management: insights from a 25-year case study in Utah and Wyoming. Rangeland Ecol Manage. 2015; 68: 375–82.

[55] Garton EO, Connelly JW, Horne JS, Hagen CA, Moser A, Schroeder MA. Greater sage-grouse population dynamics and probability of persistence In Knick ST, Connelly JW, editors. Greater sage-grouse: ecology and conservation of a landscape species and its habitats. Studies in Avian Biology 38. Berkeley: University of California Press; 2011 pp. 293–381.

[56] Utah Division of Wildlife Resources (UDWR). Utah Greater Sage-grouse Management Plan Publication 09–17. Salt Lake City: State of Utah, Department of Natural Resources, Division of Wildlife Resources; 2009. 94 pps.

[57] Manly BFJ, McDonald LL, Thomas DL, McDonald TL, Erickson WP. Resource selection by animals: statistical design and analysis for field studies. Boston: Kluwer Academic; 2002.

[58] Johnson DH. The comparison of usage and availability measurements for evaluating resource preference. Ecology. 1980; 61: 65–71.

[59] Connelly JW, Rinkes ET, Braun CE. Characteristics of greater sage-grouse habitats In Knick ST, Connelly JW, editors. Greater sage-grouse: ecology and conservation of a landscape species and its habitats. Studies in Avian Biology 38. Berkeley: University of California Press; 2011 pp 69–83.

[60] Knick ST, Hanser SE, Preston KL. Modeling ecological minimum requirements for distribution of greater sage-grouse leks: Implications for population connectivity across their western range, U.S.A. Ecol Evol. 2013; 3: 1539–1551. 10.1002/ece3.557 23789066 PMC3686190

[61] Lammers WM, Collopy MW. Effectiveness of avian predator perch deterrents on electric transmission lines. J Wildlife Manage. 2007; 71: 2752–2758.

[62] Prather PR, Messmer TA. Raptor and corvid response to power distribution line perch deterrents in Utah. J Wildlife Manage. 2010; 74: 796–800.

[63] Schroeder MA, Robb LA. Fidelity of greater sage grouse *Centrocercus urophasianus* to breeding areas in a fragmented landscape. Wildlife Biol. 2003; 9: 291–299.

[64] U.S. Bureau of Land Management (BLM). National Sage-Grouse Habitat Conservation Strategy. Washington, DC: U.S. Department of Interior; 2004.

[65] Rowland MN. Effects of management practices on grassland birds: Greater Sage-grouse. Jamestown: Northern Prairie Wildlife Research Center; 2004. 47 pps.

[66] Braun CE, Britt T, Wallestad RO. Guidelines for maintenance of sage grouse habitats. Wildlife Soc Bull. 1977; 5: 99–106.

[67] Connelly JW, Hagen CA, Schroeder MA. Characteristics and dynamics of greater sage-grouse populations In Knick ST, Connelly JW, editors. Greater sage-grouse: ecology and conservation of a landscape species and its habitats. Studies in Avian Biology 38. Berkeley: University of California Press; 2011 pp. 53–67.

[68] U.S. Bureau of Land Management (BLM). A report on national greater sage-grouse conservation measures Sage-grouse national technical team report. Washington, DC: U.S. Department of Interior; 2011. 74 pps.

[69] Wyoming Executive Order (Wyoming EO). Greater Sage-grouse Core Area Protection Executive Order 2015–4. 2015. 45pp.

[70] Beck JL, Mitchell DL, Maxfield BD. Changes in the distribution and status of sage-grouse in Utah. West N Amer Nat. 2003; 63: 203–214.

[71] Connelly JW, Knick ST, Schroeder MA, Stiver SJ. Conservation assessment of greater sage-grouse and sagebrush habitats. Unpublished Report. Western Association of Fish and Wildlife Agencies; 2004.

[72] Sandford CP, Kohl MT, Messmer TA, Cook A, Wing BR. Greater sage-grouse resource selection drives reproductive fitness under a conifer removal strategy. Rangeland Ecol Manag. 2017; 70: 59–67.

[73] Dahlgren DK, Messmer TA, Koons DN. Achieving better estimates of greater sage-grouse chick survival in Utah. J Wildlife Manage. 2010; 74: 1286–1294.

[74] Messmer, TA. Lessons learned from the greater sage-grouse: challenges and emerging opportunities for agriculture and rural communities. Policy Brief 6. National Agricultural and Rural Development Policy Center. 2013. Michigan State University, East Lansing, Michigan. USA. Available from: http://utahcbcp.org/files/uploads/publications/PolicyBrief_SageGrouse.pdfx

