# Correction: The effects of electric power lines on the breeding ecology of greater sage-grouse

**DOI:** 10.1371/journal.pone.0213669

**Published:** 2019-03-06

**Authors:** Michel T. Kohl, Terry A. Messmer, Benjamin A. Crabb, Michael R. Guttery, David K. Dahlgren, Randy T. Larsen, Shandra N. Frey, Sherry Liguori, Rick J. Baxter

There are errors in the Abstract. The correct Abstract is as follows: Anthropogenic infrastructure can negatively affect wildlife through direct mortality and/or displacement behaviors. Some tetranoids (grouse spp.) species are particularly vulnerable to tall anthropogenic structures because they evolved in ecosystems void of vertical structures. In western North America, electric power transmission and distribution lines (power lines) occur in sagebrush (Artemisia spp.) landscapes within the range of the greater sage-grouse (Centrocercus urophasianus; sage-grouse). The U.S. Fish and Wildlife Service recommended using buffer zones near leks to mitigate the potential impacts of power lines on sage-grouse. However, recommended buffer distances are inconsistent across state and federal agencies because data are lacking. To address this, we evaluated the effects of power lines on sage-grouse breeding ecology within Utah, portions of southeastern Idaho, and southwestern Wyoming from 1998–2013. Overall, power lines negatively affected lek trends up to a distance of 2.3 km. Power lines did not affect lek persistence. Female sage-grouse avoided transmission lines during the nesting and brooding seasons at distances up to 1.1 and 1.2 km, respectively. Nest and brood success were negatively affected by transmission lines, however no maximum effect distance was identified. Distribution lines did not appear to affect sage-grouse habitat selection or reproductive fitness. Our analyses demonstrated the value of sagebrush cover in mitigating potential power line impacts. Managers can minimize the effects of new transmission power lines by placing them in existing anthropogenic corridors and/or incorporating buffers at least 2.3 km from active leks. Given the uncertainty we observed in our analyses regarding sage-grouse response to distribution lines coupled with their role in providing electric power service directly to individual consumers, we recommend that buffers for these power lines be considered on a case-by-case basis. Micrositing to avoid important habitats and habitat reclamation may reduce the potential impacts of new power line construction.

Fig 4 is incorrect. The authors have provided a corrected version here.

**Fig 4:**
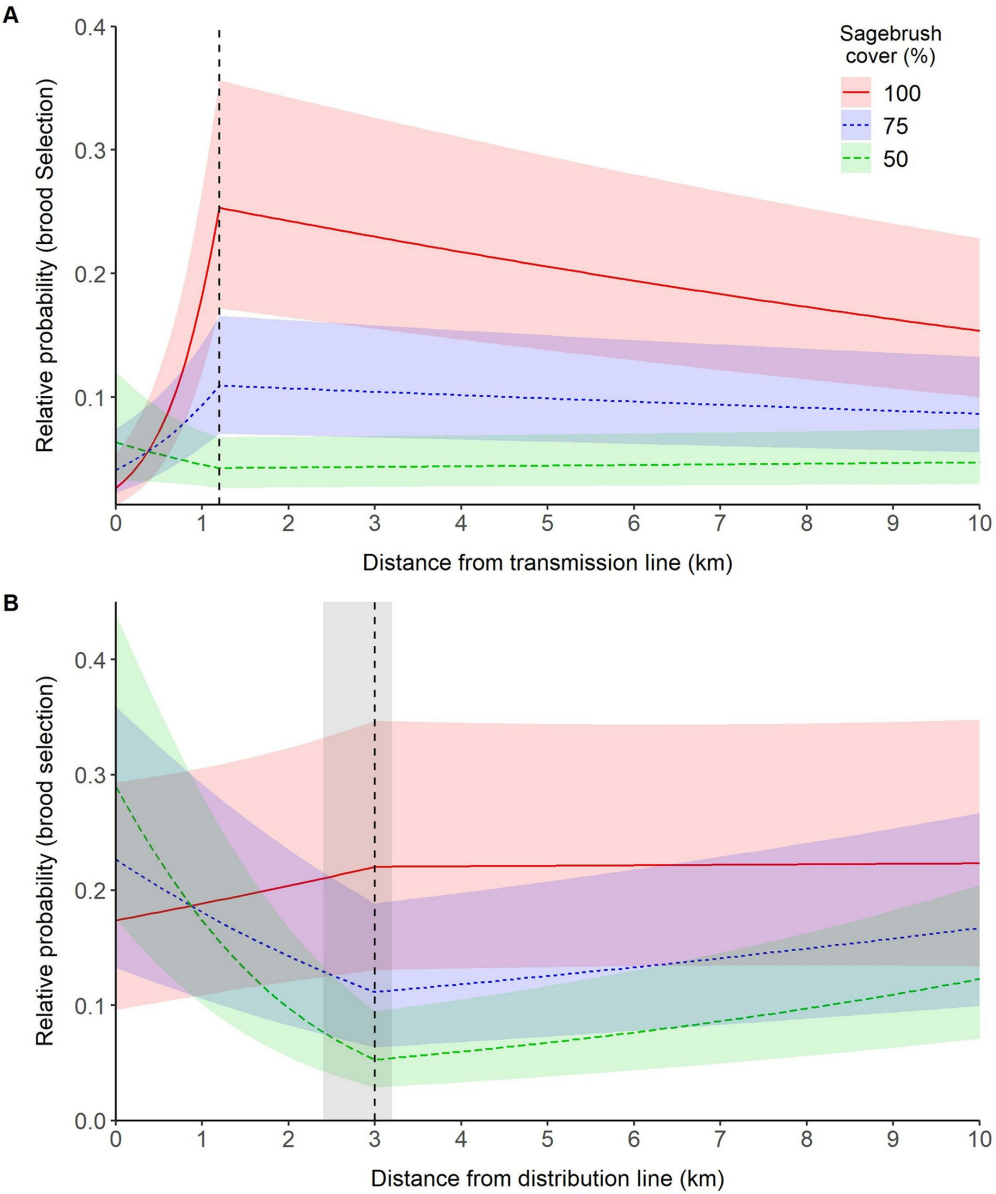
Effect of power lines on the relative probability of greater sage-grouse (*Centrocercus urophasianus*; sage-grouse) brood site selection in Utah, portions of southeastern Idaho, and southwestern Wyoming, USA, 1998–2013. Lines are population-averaged fitted values from the best-fit GLMM (S4 Appendix) describing the effects of transmission lines (A) and distribution lines (B) on sage-grouse brood site selection. The vertical dashed line identifies the response threshold at which sage grouse response changes. The shaded areas highlight uncertainty (ΔAICc < 2) around the location of the response threshold (no uncertainty was recorded for distance from transmission lines).
