# Supplementary material for: The effects of electric power lines on the breeding ecology of greater sage-grouse
Source: PLoS One. 2019 Jan 30;14(1):e0209968. doi: 10.1371/journal.pone.0209968 (PMC6353545; doi:10.1371/journal.pone.0209968)

**S1 Appendix.**

Supplemental information pertaining to the relationship between greater sage-grouse (*Centrocercus urophasianus*) elevational gain and Julian date during the brooding season. This appendix includes graphical representations of the raw data, as well as model selection tables and coefficient estimates for each best-fit model.

*Elevation gain across brooding season*

**Fig 1. Graphical representation of all telemetry locations for brooding female greater sage-grouse (*Centrocercus urophasianus*) across eight study areas in which broods were monitored.** Data represents the unmodelled population-level relationship between elevation and Julian date (i.e., we did not account for individual-level brood behaviors). From this information, no clear trend is visible from exploration of the raw data. The blue line represents the linear relationship between elevation and Julian date for each study area with a 95% confidence interval.

**
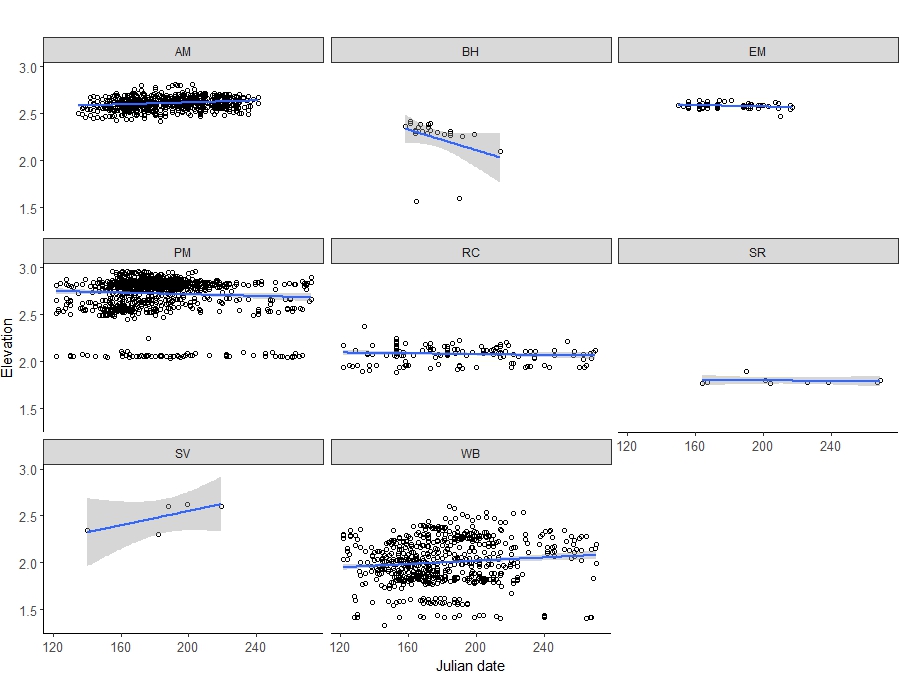
**

**Table 1. Model selection results for generalized linear model describing the relationship between greater sage-grouse (*Centrocercus urophasianus*) relative probability of use and elevation and Julian date in Utah, portions of southeastern Idaho, and southwestern Wyoming, USA, 1998-2013.** We did not consider the univariate or additive effect of Julian date because it has no spatial references, and as such, is only considered influential via its interaction with elevation. Number of parameters, AICc, and differences in AICc compared to the best scoring model (ΔAIC) are given for each model. The best model is in bold face.


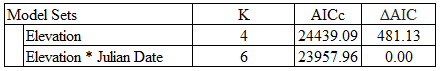


**Table 2. Best-fit generalized linear model for the effects of elevation and Julian date on the relative probability of use by female greater sage-grouse (*Centrocercus urophasianus*) during the brooding season in Utah, portions of southeastern Idaho, and southwestern Wyoming, USA, 1998-2013.**


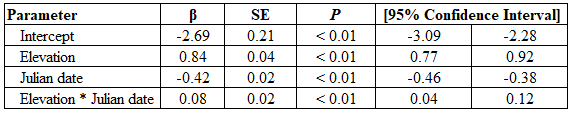


**Fig 2. Effect of elevation on the relative probability of brood site selection during the brooding period of greater sage-grouse (*Centrocercus urophasianus*; sage-grouse) in Utah, portions of southeastern Idaho, and southwestern Wyoming, USA, 1998-2013.** Lines are population-averaged fitted values from the best-fit GLMM (S1 Appendix – Table 1). Although sage-grouse prefer higher elevation locations during the brooding period, the relative probability of selection decreases as the season progresses suggesting they are selecting for lower elevation sites.


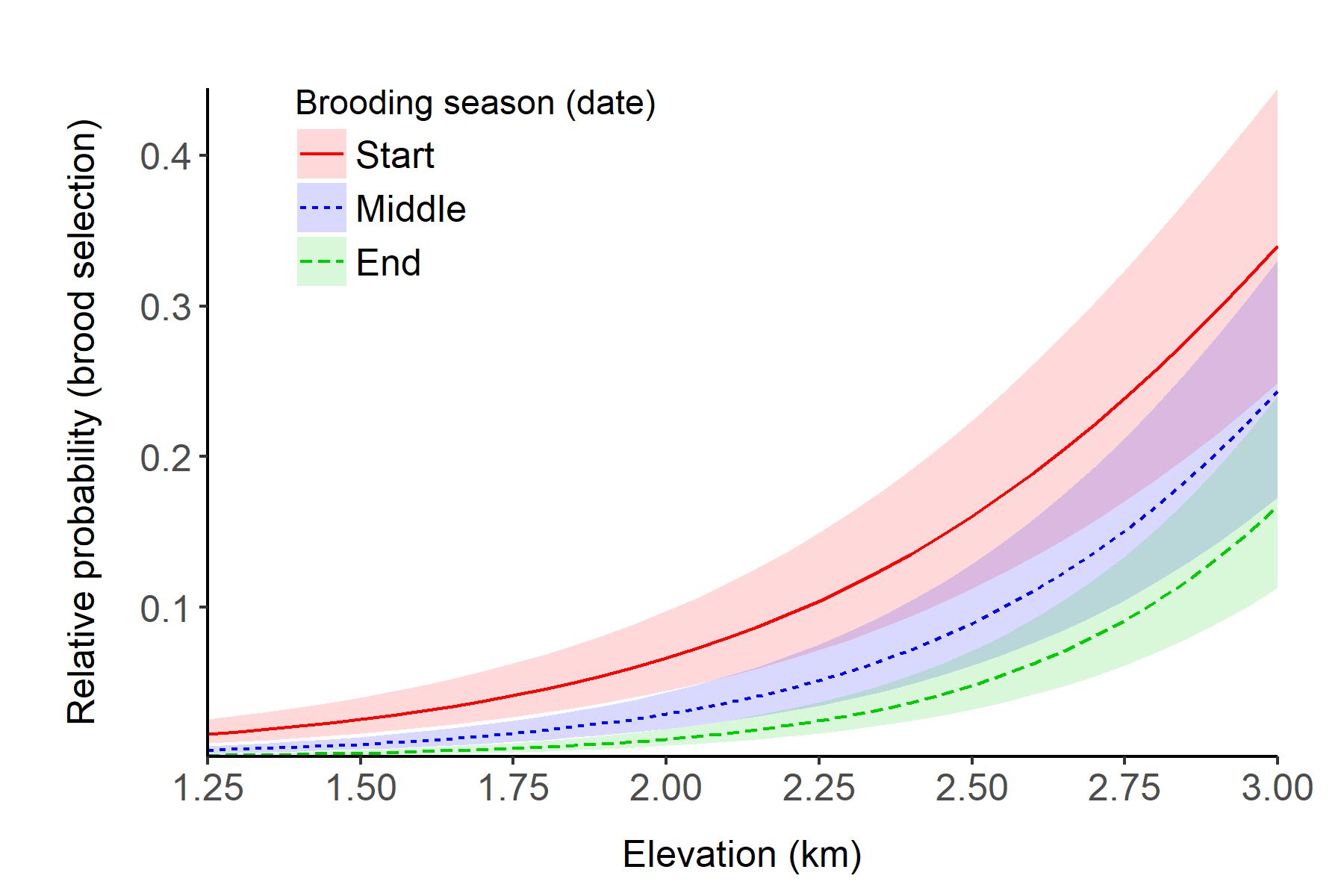

Supplement: S1 Appendix — (DOCX) [file pone.0209968.s001.docx]
