# Supplementary material for: The effects of electric power lines on the breeding ecology of greater sage-grouse
Source: PLoS One. 2019 Jan 30;14(1):e0209968. doi: 10.1371/journal.pone.0209968 (PMC6353545; doi:10.1371/journal.pone.0209968)

**S2 Appendix**

Supplemental information pertaining to the relationship between greater sage-grouse (*Centrocercus urophasianus*) lek trend or persistence and distance to power lines. This appendix includes information on model selection tables and coefficient estimates for each best-fit model.

**Lek Trend**

**Table 1. Model selection results for generalized linear model describing the relationship between greater sage-grouse (*Centrocercus urophasianus*) lek trends and distance to power lines in Utah, 1998-2013.** Variables DistP1 and DistP2 contain a linear spline for distance to power line at the indicated threshold. The simple linear model (DistP) includes no threshold. Number of parameters, AICc, and differences in AICc compared to the best scoring model (ΔAIC) are given for each model. The best model for each distance to power lines is in bold face. Model uncertainty around the threshold (ΔAIC < 2.0) is identified by the competing models; models that extend beyond the uncertainty range are not shown.


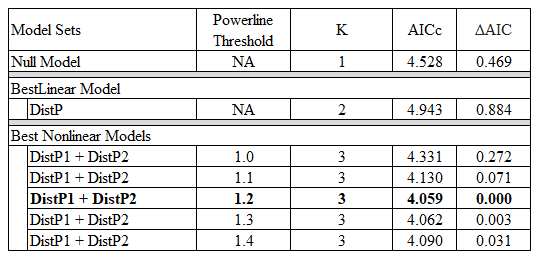


**Table 2. Best-fit generalized linear model for the effects of distance to power lines on greater sage-grouse (*Centrocercus urophasianus*) lek trend in Utah, 1998-2013.** Variables DistP1 and DistP2 are the slopes before and after the threshold identified in the model selection approach presented above.


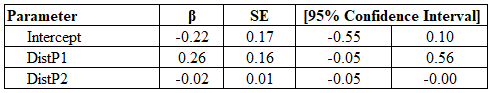


**Lek Persistence**

**Table 3. Model selection results for generalized linear model describing the relationship between greater sage-grouse lek (*Centrocercus urophasianus*) persistence and distance to power lines in Utah, 1998-2013.** Variables DistP1 and DistP2 contain a linear spline for distance to power line at the indicated threshold. The simple linear model (DistP) includes no threshold. Number of parameters, AICc, and differences in AICc compared to the best scoring model (ΔAIC) are given for each model. The best model for each distance to power lines is in bold face.


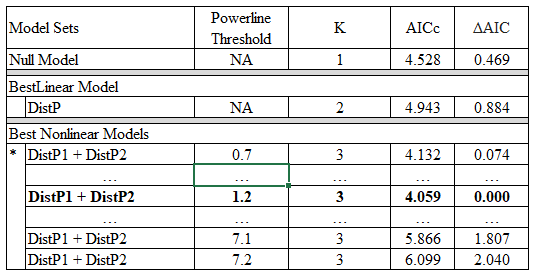


**Table 4. Model uncertainty around the threshold (ΔAIC < 2.0) is identified by the competing models; models that extend beyond the uncertainty range are not shown.** The asterisk identifies the point at which subsequent threshold models would have included knots that did not fall within the 5% - 95% quartiles of the data. For example, a model with a threshold at 0.7 km would estimate the relationship between distance to power lines and lek persistence using 3.6 % of the available data, whereas the model that included a threshold at 0.8 km included 5.4 % of the data. This was done to minimize outlier effects. In such cases, model uncertainty was not estimated below 0.8 km.


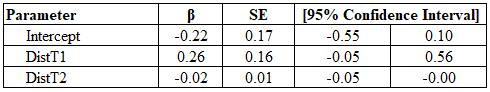

Supplement: S2 Appendix — (DOCX) [file pone.0209968.s002.docx]
