# Supplementary material for: The effects of electric power lines on the breeding ecology of greater sage-grouse
Source: PLoS One. 2019 Jan 30;14(1):e0209968. doi: 10.1371/journal.pone.0209968 (PMC6353545; doi:10.1371/journal.pone.0209968)

**S4 Appendix**

Supplemental information pertaining to the relationship between greater sage-grouse (*Centrocercus urophasianus*) brood site selection and the distance to transmission and distribution power lines. This appendix includes information on model selection tables and coefficient estimates for each best-fit model.

**Brood Site Selection – Transmission Lines**

**Table 1. Model selection results for generalized linear mixed model describing the relationship between greater sage-grouse (*Centrocercus urophasianus*) brood site selection and distance to transmission lines in Utah, portions of southeastern Idaho, and southwestern Wyoming, USA, 1998-2013.** Variables DistT1 and DistT2 contain a linear spline for distance to a transmission line at the indicated threshold. The variable Sage identifies the percent sagebrush cover covariate. The simple linear model (DistT) includes no threshold. Number of parameters, AICc, and differences in AICc compared to the best scoring model (ΔAIC) are given for each model. The best model for each distance to power lines is in bold face.


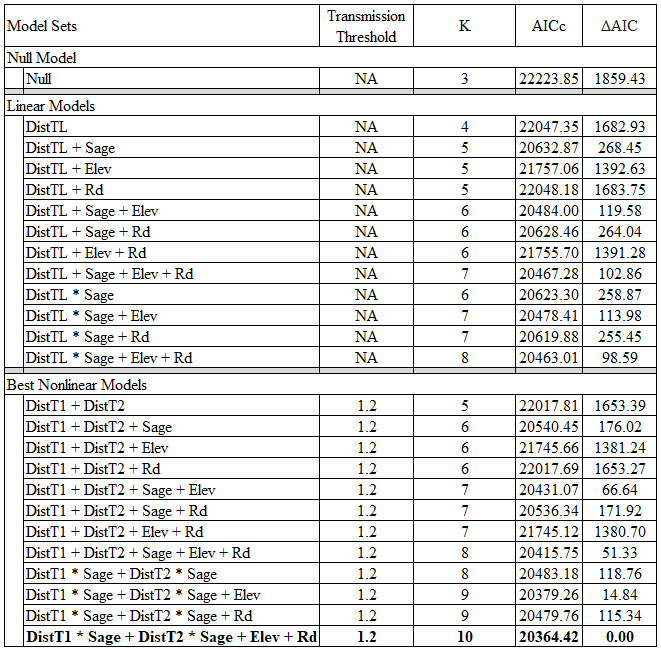


**Table 2. Model uncertainty around the threshold (ΔAIC < 2.0) is identified by the competing models; models that extend beyond the uncertainty range are not shown.**


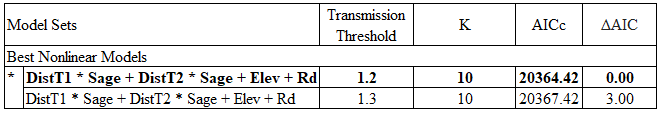


**Table 3. Best-fit generalized linear model for the effects of distance to transmission lines on greater sage-grouse (*Centrocercus urophasianus*) nest site selection in Utah, 1998-2013.** Variables DistP1 and DistP2 are the slopes before and after the threshold identified in the model selection approach presented above.


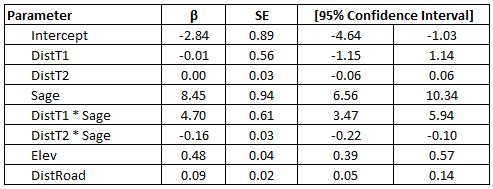


**Brood Site Selection – Distribution Lines**

**Table 4. Model selection results for generalized linear mixed model describing the relationship between greater sage-grouse (*Centrocercus urophasianus*) nest site selection and distance to distribution lines in Utah, portions of southeastern Idaho, and southwestern Wyoming, USA, 1998-2013.** Variables DistT1 and DistT2 contain a linear spline for distance to a distribution line at the indicated threshold. The variable Sage identifies the percent sagebrush cover covariate. The simple linear model (DistT) includes no threshold. Number of parameters, AICc, and differences in AICc compared to the best scoring model (ΔAIC) are given for each model. The best model for each distance to power lines is in bold face.


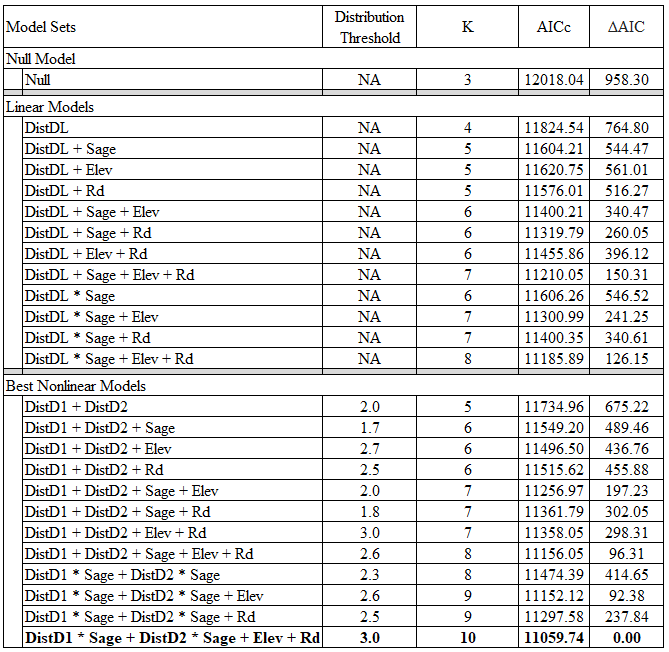


**Table 5. Model uncertainty around the threshold (ΔAIC < 2.0) is identified by the competing models; models that extend beyond the uncertainty range are not shown.**


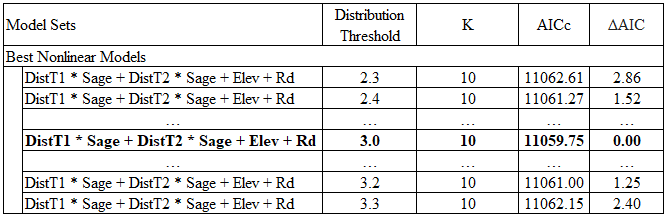


**Table 6. Best-fit generalized linear model for the effects of distance to transmission lines on greater sage-grouse (*Centrocercus urophasianus*) nest site selection in Utah, 1998-2013.** Variables DistP1 and DistP2 are the slopes before and after the threshold identified in the model selection approach presented above.
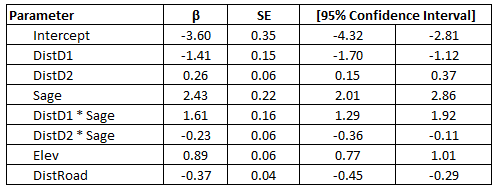

Supplement: S4 Appendix — (DOCX) [file pone.0209968.s004.docx]
