# Supplementary material for: The effects of electric power lines on the breeding ecology of greater sage-grouse
Source: PLoS One. 2019 Jan 30;14(1):e0209968. doi: 10.1371/journal.pone.0209968 (PMC6353545; doi:10.1371/journal.pone.0209968)
Supplement: S7 Appendix — (DOCX) [file pone.0209968.s007.docx]

**S7 Appendix.**

**Table 1. Road and power line correlations.** Kilometers (km) of ancillary roads by type associated with electric transmission and distribution power lines within a 10 km buffer of a greater sage-grouse (*Centrocercus urophasianus*) nest and brood locations in Utah and portions of southeastern Idaho, and southwestern Wyoming, USA, 1998-2013. *

|  | **Km within 100 m of roads** | | **Km within 0.5 km of roads** | |
| --- | --- | --- | --- | --- |
| **Road Type** | **Distribution** | **Transmission** | **Distribution** | **Transmission** |
| Any road | 1037.7 | 909.6 | 1427.7 | 1481.4 |
| Highway | 30.9 | 39.0 | 32.7 | 40.8 |
| Non-highway paved roads | 996.3 | 855.0 | 1414.5 | 1439.1 |
| Non-paved roads | 20.7 | 37.2 | 87.9 | 140.7 |
| Other roads | 57.9 | 28.8 | 254.4 | 164.1 |

* When we considered distribution lines within 10 km of a sage-grouse location, 69% were within 100 m of a road and 95% were within 0.5 km of a road. When we considered transmission lines within 10 km of a sage-grouse location, 54% were within 100 m of a road and 87% were within 0.5 km of a road.
